# Immunogenicity and safety of different schedules of the meningococcal ABCWY vaccine, with assessment of long-term antibody persistence and booster responses – results from two phase 2b randomized trials in adolescents

**DOI:** 10.1080/21645515.2021.1968214

**Published:** 2021-09-28

**Authors:** Timo Vesikari, Jerzy Brzostek, Anitta Ahonen, Marita Paassilta, Ewa Majda-Stanislawska, Leszek Szenborn, Miia Virta, Robert Clifford, Teresa Jackowska, Murray Kimmel, Ilaria Bindi, Pavitra Keshavan, Paola Pedotti, Daniela Toneatto

**Affiliations:** a Nordic Research Network Oy, Tampere, Finland; b Health Care Establishment in Debica, Infectious Diseases Outpatient Clinic, Debica, Poland; c Vaccine Research Center, Tampere University, Tampere, Finland; d Tampere University and Tampere University Hospital, Tampere, Finland; e Department of Pediatric Infectious Diseases, Medical University of Lodz, Lodz, Poland; f Department of Pediatric Infectious Diseases, Wroclaw Medical University, Wroclaw, Poland; g Coastal Pediatric Research, Charleston, SC, USA; h Department of Pediatrics, The Medical Centre of Postgraduate Education, Warsaw, Poland; i Optimal Research LLC, Melbourne, FL, USA; j Gsk, Siena, Italy; k Gsk, Amsterdam, The Netherlands

**Keywords:** MenABCWY vaccine, non-inferiority, persistence, booster, adolescents

## Abstract

The meningococcal serogroup B (MenB) protein vaccine, 4CMenB, combined with MenA, MenC, MenW and MenY polysaccharide-protein conjugates for a pentavalent MenABCWY vaccine, can potentially protect against most causative agents of invasive meningococcal disease worldwide. Two phase 2b, randomized, multicenter studies were conducted (NCT02212457, NCT02946385) to assess the immunogenicity and safety of the MenABCWY vaccine as well as antibody persistence and response to a booster dose 2 years after the last vaccination, compared to 4CMenB vaccination. Participants (10 − 18 years), randomized (3:3:2:2:2:2), received the 4-component 4CMenB vaccine according to a 0–2 month (M) schedule or MenABCWY according to a 0–2, 0–6, 0-2-6, 0–1, or 0–11 M schedule. All participants received 5 injections (at M0, M1, M2, M6 and M12) with either the study vaccines or placebo/hepatitis A vaccine. Follow-on participants (4CMenB-0-2, MenABCWY-0-2, MenABCWY-0-6 and MenABCWY-0-2-6 groups) received one dose of either 4CMenB (4CMenB-0-2 group) or MenABCWY and newly enrolled, age-matched, meningococcal vaccine-naïve adolescents (randomized 1:1) received 2 doses (0–2 M) of either 4CMenB or MenABCWY. MenABCWY vaccination was immunogenic against MenB test strains. Non-inferiority for all 4 components of the 4CMenB vaccine could not be demonstrated for the 0–2 M schedule. Antibodies persisted up to 2 years post-MenABCWY vaccination and a booster dose induced an anamnestic response as higher titers were observed in follow-on participants compared to the first-dose response in vaccine-naïve participants. MenABCWY had a clinically-acceptable safety profile, not different from that of 4CMenB.

## Introduction

*Neisseria meningitidis* causes invasive meningococcal disease (IMD) that may rapidly progress from early symptoms to death in 24–48 hours. Even with adequate antibiotic therapy, case-fatality rates remain high (8%–15%) and more than 10% of survivors will suffer from severe sequelae such as limb amputations, hearing loss and neurological damage.^1^ This makes IMD an important health concern despite the fact that IMD incidence rates are low in most industrialized countries. In 2017, an incidence rate of 0.11 cases per 100 000 people was reported in the United States (US) and the incidence rate in Europe in 2016 was 0.6 cases per 100 000 people.^2,3^ The disease occurs most frequently among infants and toddlers, followed by a second peak in young adults/adolescents and, in some countries, by another peak in older adults.^4–6^

Six meningococcal serogroups (MenA, MenB, MenC, MenW, MenX and MenY) are responsible for nearly all IMD worldwide, but serogroup distribution changes continuously over time and can vary substantially from one geographical region to another.^1,7^ While in the African meningitis belt MenA has accounted for most of the meningococcal disease cases before mass vaccination campaigns and vaccine introduction, recent outbreaks are primarily caused by MenC, MenW and MenX.^7,8^ In Europe, North America, Australia, and New Zealand, meningococcal disease is caused by MenB, MenC, MenW and MenY, with MenB being the most prevalent.^7,9–11^

The IMD incidence has decreased following inclusion of meningococcal vaccines in national immunization programs targeting infants or adolescents, with the latter being the most frequent asymptomatic carriers.^12–14^ During the last decades, several monovalent polysaccharide-protein conjugate vaccines against MenC and MenA, quadrivalent MenACWY-conjugate vaccines, and protein vaccines against MenB have successfully been introduced.^15^ A MenACWY+MenB formulation can potentially protect against virtually all-cause IMD by addressing the high prevalence of MenB in developed countries while controlling IMD caused by MenACWY. In addition, a combined, pentavalent MenABCWY vaccine would facilitate vaccine schedules, particularly in adolescents.

The quadrivalent meningococcal conjugate vaccine MenACWY-CRM (*Menveo*, GSK) has demonstrated immunogenicity and an acceptable safety profile when administered alone or in combination with other routine vaccines, including the 4-component MenB vaccine (4CMenB, *Bexsero*, GSK).^16–23^ 4CMenB is licensed for use as a 2-dose schedule in individuals from 2 months of age in Europe and 10–25 years of age in the US.^24,25^ The vaccine has been shown to be immunogenic, with real-life effectiveness demonstrated in the United Kingdom following vaccine introduction in the national immunization program.^26–32^ A MenACWY+MenB vaccine combining MenACWY-CRM and 4CMenB (MenABCWY) has been shown to be immunogenic and well tolerated when administered to adolescents and young adults, but remains under investigation.^33–37^ The present study compared the immune responses to 4CMenB antigens after vaccination with the pentavalent MenABCWY vaccine and the 4CMenB vaccine alone. MenACWY-CRM was not used as a comparator in this study, but immune responses to MenA, MenC, MenW and MenY were also measured.

The trial aimed to demonstrate non-inferiority of MenABCWY to 4CMenB vaccination and to assess five different MenABCWY schedules in terms of immune responses against 4 MenB antigen-specific indicator strains and serogroup A, C, W and Y polysaccharide antigens. The study was extended to assess antibody persistence at 24 months post-primary vaccination and response to a MenABCWY booster dose.

## Patients and methods

### Study design and participants

**Figure 1.**
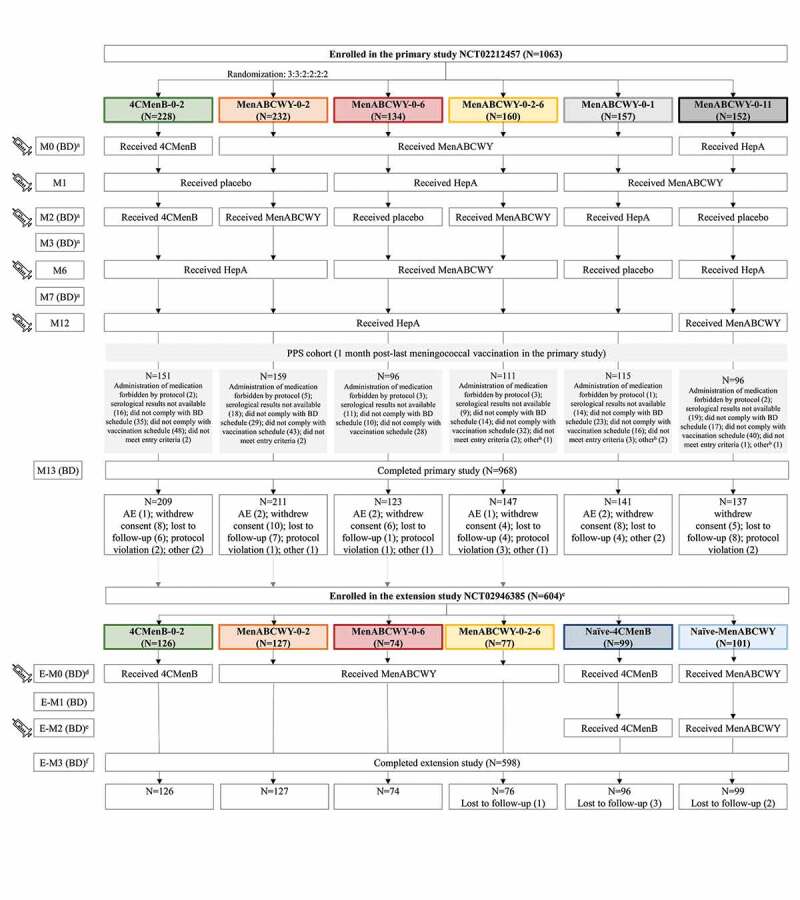
Participant flow chart and study interventions.

The primary study, a phase 2b, randomized, controlled, observer-blind study (NCT02212457) was conducted in 32 centers in Finland, Poland and the US, between August 2014 and March 2016. Healthy adolescents, 10–18 years of age at the time of first vaccination, were enrolled in the study if they gave their informed consent/assent, were considered as able and willing to comply with protocol requirements and had a negative urine pregnancy test (for females of childbearing age). Adolescents were not eligible for enrollment if they had any history of meningococcal or hepatitis A (HepA) vaccination (used in the study as active control), had current or previous, confirmed or suspected disease by *N. meningitidis* or household contact with and/or intimate exposure to individuals with laboratory-confirmed *N. meningitidis* infection within 60 days from enrollment. Participants were randomized in a 3:3:2:2:2:2 ratio to 1 of 6 groups to either receive 4CMenB according to a 0–2 month (M) schedule, or MenABCWY according to a 0–2, 0–6, 0-2-6, 0–1, or 0–11 M schedule. All participants received 5 injections (at M0, M1, M2, M6 and M12), consisting either of the study vaccines or placebo/HepA vaccine (Figure 1).

N, number of participants; 4CMenB, 4-component meningococcal serogroup B vaccine; MenABCWY, pentavalent meningococcal serogroup A, B, C, W and Y vaccine; M, study month in the primary study; BD, blood draw; HepA, hepatitis A vaccine; PPS, per-protocol set; AE, adverse event; E-M, study month in the extension study.^a^ A minimum of 23 days between the study vaccination and the post-vaccination blood draw were required.^b^ Vaccine not administered, but subject number allocated.^c^ The extension study was not conducted in the US.^d^ Two blood samples were collected in follow-on participants: pre-vaccination and at 5 days post vaccination.^e^ The blood sample in the naïve group was collected at 5 days post vaccination. No blood samples were collected in follow-on participants.^f^ No blood samples were collected in follow-on participants.

Randomization was performed using an interactive response technology (IRT) system. Participants were stratified in 2 age groups (10–15 and 16–18 years) and randomized into 1 of the 6 groups. Block randomization was used and within each block (used only by one site) the treatments were balanced. Both the study personnel responsible for the evaluation of any study endpoint and the participants or their parents/legal guardians were blinded to the treatment throughout the study. Designated medical personnel that did not participate in the evaluation of study endpoints oversaw vaccine preparation and administration. For US sites only, a restricted unblinding process was carried out after completion of the last visit/contact in the primary study to identify participants who had received an ACWY-containing vaccine. The unblinding was carried out because the US Advisory Committee on Immunization Practices recommends routine administration of MenACWY at 11 or 12 years of age, with a booster dose at 16 years of age and confirmation of vaccination is required in many states for school and military purposes.^38^ Unblinding was performed by unblinded medical personnel using the IRT system and a vaccination certificate was provided to all US subjects who did not terminate early, indicating if they received 2 doses of 4CMenB (subjects identified during the restricted unblinding) or at least 2 doses of MenABCWY (subjects not identified during the restricted unblinding). Blinded site personnel and sponsor representatives had no access to this information.

An extension, open-label study (NCT02946385) was conducted in Finland and Poland only between November 2016 and February 2018, 2 years after the last vaccination in the primary study. Adolescents from Finland and Poland previously enrolled in one of the 4CMenB-0-2, MenABCWY-0-2, MenABCWY-0-6 and MenABCWY-0-2-6 groups and who had not received any additional meningococcal vaccine in the interim period, were eligible for enrollment in the extension study and received one dose of either 4CMenB (group 4CMenB-0-2) or MenABCWY (all other groups). In addition, age-matched (12–20 years), meningococcal vaccine-naïve adolescents were enrolled and randomized 1:1 to receive 2 doses of either 4CMenB or MenABCWY, according to a 0–2 M schedule (Figure 1).

Both studies were conducted in agreement with the International Conference on Harmonization guidelines for good clinical practice, applicable local regulations, and the Declaration of Helsinki. The protocol and the proposed informed consent/assent forms were approved by independent ethics committees in Finland and Poland or institutional review boards at each site in the US. The studies are registered at www.clinicaltrials.gov and full study protocols are available at https://www.gsk-studyregister.com/study/5786 and https://www.gsk-studyregister.com/study/5815.

### Study vaccines

The injected volume of vaccine/placebo was 0.5 mL and was injected intramuscularly in the deltoid of the non-dominant arm. For preparation of the MenABCWY vaccine, the lyophilized MenACWY powder was reconstituted with the liquid 4CMenB suspension prior to administration, as previously described.^39^ One dose of the 4CMenB vaccine contains 50 µg of recombinant MenB *Neisseria* adhesin A (NadA) protein, 50 µg of recombinant MenB Neisserial Heparin Binding Antigen (NHBA) fusion protein, 50 µg of recombinant MenB factor H binding protein (fHbp) fusion protein and 25 µg of outer membrane vesicles from MenB strain NZ98/254 measured as amount of total protein containing the porin A (PorA) P1.4. One dose of the MenABCWY vaccine contains 10 µg of MenA-CRM_197_, 5 µg of MenC-CRM_197_, 5 µg of MenW-CRM_197_ and 5 µg of MenY-CRM_197_ in addition to the components present in the 4CMenB vaccine.^25,39^ Pediatric and adult formulations of the HepA vaccine (*Havrix*, GSK) were used for participants aged ≤15 years and >16 years, respectively.^40^ The placebo was a saline solution (4.5 mg NaCl/0.5 mL).

### Objectives

In the primary study, the primary objective was to demonstrate the non-inferiority of MenABCWY to 4CMenB, administered according to a 0–2 M schedule, as measured by serum bactericidal assay using human complement (hSBA) geometric mean titers (GMTs) for MenB test strains, at 1 month after the last meningococcal vaccination. Non-inferiority was to be concluded if the lower limit (LL) of the 2-sided 95% confidence interval (CI) for the between-group ratios of GMTs (MenABCWY-0-2:4CMenB-0-2) was >0.5 for each of the 4 MenB test strains. Secondary objectives included the evaluation of safety and reactogenicity, assessment of the immunogenicity of the various MenABCWY schedules in terms of percentages of participants with hSBA titers at least equal to the assay’s lower limit of quantitation (LLOQ) and hSBA GMTs against MenB strains, MenA, MenC, MenW, and MenY.

In the extension study, the primary objective included the assessment of persistence of bactericidal antibodies at 24 months after the last meningococcal vaccination in the primary study, compared with antibody levels in age-matched meningococcal-naïve participants at enrollment, as measured by percentages of participants with hSBA titers ≥LLOQ and hSBA GMTs against MenB test strains. Secondary objectives included the assessment of MenB immune response to booster vaccination with MenABCWY or 4CMenB in follow-on participants compared with the response to a first vaccination in naïve participants, evaluation of immune responses against MenA, MenC, MenW and MenY in follow-on and naïve participants in MenABCWY groups, and evaluation of safety and reactogenicity.

### Immunogenicity assessment

Blood samples of approximately 20 mL were collected at the timepoints indicated in Figure 1. Serum bactericidal activity was measured using an automated high-throughput hSBA for 4 MenB test strains directed against each antigenic component of 4CMenB (M14459 against fHbp, 96217 against NadA, M07-0241084 against NHBA, NZ98/254 against PorA), and against capsular polysaccharides for MenA, MenC, MenW and MenY.^41^ The LLOQs were 8.0 (fHbp), 8.6 (NadA), 8.9 (NHBA), 8.2 (PorA), 22.7 (MenA), 5.2 (MenC), 39.6 (MenW), and 14.7 (MenY).

Testing was performed at Clinical Laboratory Sciences, GSK, Marburg, Germany.

The percentage of participants with hSBA titers ≥LLOQ and 4-fold titer rise, and hSBA GMTs were evaluated for MenB test strains and ACWY serogroups. The 4-fold titer rise was defined as a post-vaccination hSBA ≥4 LLOQ for participants with pre-vaccination hSBA titers <LLOQ and an increase of ≥4 times the pre-vaccination hSBA titer for participants with pre-vaccination hSBA titers ≥LLOQ. For MenA, MenC, MenW and MenY, a post-vaccination hSBA titer ≥4 was used as a correlate of protection against IMD, as accepted for MenC^42^ and extended to the other *N. meningitidis* serogroups.

### Safety assessment

Following each vaccination in both the primary and extension study, participants were observed for at least 30 minutes and any adverse events (AEs) were recorded. Solicited local and systemic AEs occurring during each 7-day post-vaccination period (from 6 hours post vaccination on day 1 up to day 7) were recorded by the participants or their parents/legal guardians using an electronic diary. Solicited AEs persisting after day 7 were recorded until resolution. Unsolicited AEs were collected during each 30-day post-vaccination period; medically attended AEs (MAAEs), AEs leading to withdrawal and serious AEs (SAEs) were collected throughout the study. AEs were graded by severity (from mild to severe) and their relationship to study vaccination was assessed by the investigators.

### Statistical analyses

A total of approximately 1050 adolescents were planned for enrollment in the primary study, to provide approximately 945 evaluable participants, when assuming a dropout rate of 10%.

For 200 evaluable participants in the 4CMenB-0-2 and MenABCWY-0-2 groups and an underlying hSBA GMT ratio of 0.9 (group MenABCWY-0-2 versus group 4CMenB-0-2, assumed based on immunogenicity results from previous trials in adolescents and young adults^37,43^), the power calculated to reject all 4 null hypotheses for the primary objective was 89.4%.

The targeted sample size in the extension study was 100 participants per group, considering that ≥652 individuals from Finland and Poland who participated in the primary study were eligible for enrollment. One hundred age-matched adolescents and young adults were targeted for enrollment in each of the meningococcal vaccine-naïve groups.

The primary objective of the primary study was evaluated using the per-protocol set (PPS), which included all participants who received a study vaccination, provided evaluable serum samples at the relevant timepoints and did not have protocol deviations. All other immunogenicity analyses (the extension study included) were conducted on the full analysis set (FAS) at each timepoint. Safety analyses were performed for all exposed participants with available safety data.

The hSBA titers were log_10_ transformed and the GMTs, with their associated 2-sided 95% CIs, were calculated by exponentiating the corresponding log-transformed means and their 95% CIs obtained from the analysis of variance (ANOVA) model, with study center included as an independent variable. Immunogenicity endpoints, based on percentages of participants, were provided with the associated 95% Clopper-Pearson CIs.

The between-group ratio of hSBA GMTs at 1 month after the last meningococcal vaccination in the primary study (group MenABCWY-0-2 versus group 4CMenB-0-2), adjusted for pre-vaccination titer and region, was calculated by exponentiating the between-group difference in the last square means of the log-transformed titers and the corresponding 95% CIs.

## Results

### Demographic characteristics

In total, 1063 adolescents were enrolled in the primary study, of whom 968 completed the study. Six hundred and four adolescents and young adults were enrolled in the extension study, 2 years later, of whom 598 completed the study (Figure 1).

The average age of participants was 14.4 ± 3.1 years in the primary study and 16.7 ± 3.0 years in the extension study. Overall, more females enrolled in both studies and most participants (>95%) were white (Table 1).

**Table 1.** Demographic and baseline characteristics (enrolled set)

		4CMenB 0–2	MenABCWY 0–2	MenABCWY 0–6	MenABCWY 0-2-6	MenABCWY 0-1	MenABCWY 0-11			Total
Primary study	N	228	232	134	160	157	152			1063
	Age, mean±SD (years)	14.5 ± 3.1	14.2 ± 3.2	14.4 ± 3.1	14.3 ± 3.2	14.4 ± 3.0	14.5 ± 3.1			14.4 ± 3.1
	Age group, n (%)									
	10–15 years	131 (57)	134 (58)	79 (59)	93 (58)	91 (58)	86 (57)			614 (58)
	16–18 years	97 (43)	98 (42)	55 (41)	67 (42)	66 (42)	66 (43)			449 (42)
	Weight, mean±SD (kg)	59.1 ± 17.6	56.6 ± 18.3^a^	57.9 ± 15.7	58.5 ± 19.6	57.3 ± 17.6	57.3 ± 16.4			57.8 ± 17.6^a^
	Height, mean±SD (cm)	162.7 ± 13.2	160.7 ± 14.3	162.1 ± 13.0	161.5 ± 14.6^a^	162.4 ± 13.1	162.5 ± 13.5			161.9 ± 13.6^a^
	Female, n (%)	130 (57)	119 (51)	76 (57)	96 (60)	101 (64)	89 (59)			611 (57)
	White, n (%)^b^	214 (94)	221 (95)	129 (96)	151 (94)	148 (94)	142 (93)			1005 (95)
		4CMenB 0–2	MenABCWY 0–2	MenABCWY 0-6	MenABCWY 0-2-6			Naïve-4CMenB	Naïve-MenABCWY	Total
Extension study	N	126	127	74	77			99	101	604
	Age, mean±SD (years)	16.8 ± 3.1	16.6 ± 3.2	17.3 ± 3.0	17.1 ± 3.0			16.2 ± 2.8	16.5 ± 2.7	16.7 ± 3.0
	Age group, n (%)									
	12–17 years	73 (58)	73 (57)	40 (54)	42 (55)			57 (58)	57 (56)	342 (57)
	18–22 years	53 (42)	54 (43)	34 (46)	35 (45)			42 (42)	44 (44)	262 (43)
	Weight, mean±SD (kg)	63.9 ± 15.5	63.8 ± 16.9	67.0 ± 14.9	66.2 ± 20.4			61.6 ± 14.0	65.4 ± 16.0	64.4 ± 16.3
	Height, mean±SD (cm)	166.9 ± 9.5	166.1 ± 9.7	169.1 ± 9.8	167.4 ± 11.0			167.0 ± 9.6	167.8 ± 9.3	167.2 ± 9.8
	Female, n (%)	82 (65)	69 (54)	44 (59)	50 (65)			58 (59)	60 (59)	363 (60)
	White, n (%)^b^	126 (100)	127 (100)	73 (99)	75 (97)			97 (98)	100 (99)	598 (99)

4CMenB, 4-component meningococcal serogroup B vaccine; MenABCWY, pentavalent meningococcal serogroup A, B, C, W and Y vaccine; N, number of participants; n (%), number (percentage) of participants in each category; SD, standard deviation.

^a^Values are given for (N-1) participants.

^b^Other included Asian, Black or African American ethnicities for the primary study and Asian, American Indian or Alaska native for the extension study.

### Immunogenicity

#### Immune response to MenB antigens

In the primary study, 1 month after the second meningococcal vaccine dose, the between-group ratios of GMTs (group MenABCWY-0-2 versus group 4CMenB-0-2) in the PPS ranged from 0.49 (for PorA) to 0.74 (for fHbp); the LL of the associated 95% CI were >0.5 for all antigens except PorA (Table 2). Therefore, the pre-defined criterion for non-inferiority was met for fHbp, NHBA and NadA, but not for PorA, and non-inferiority could not be demonstrated for this schedule.

**Table 2.** hSBA geometric mean titers and between group ratios (MenABCWY-0-2:4CMenB-0-2), at 1 month post-last meningococcal vaccination in the primary study (per-protocol set)

	4CMenB-0-2		MenABCWY-0-2		Between groups
	N	GMT (95% CI)	N	GMT (95% CI)	GMT (95% CI)
*fHbp*					
Baseline	147	1.31 (1.09–1.57)	155	1.28 (1.07–1.53)	0.98 (0.81–1.19)
1 M post-second dose	150	15.78 (12–22)	158	11.64 (8.61–16)	0.74 (**0.53**–1.02)
*NadA*					
Baseline	143	2.36 (1.70–3.28)	152	2.93 (2.13–4.03)	1.24 (0.89–1.74)
1 M post-second dose	149	229.29 (179–294)	156	150.82 (118–192)	0.66 (**0.51**–0.85)
*NHBA*					
Baseline	148	2.16 (1.61–2.89)	151	2.12 (1.60–2.82)	0.98 (0.73–1.33)
1 M post-second dose	150	11.56 (8.86–15)	154	8.19 (6.31–11)	0.71 (**0.54**–0.94)
*PorA*					
Baseline	147	1.15 (0.95–1.39)	154	1.27 (1.06–1.53)	1.11 (0.91–1.34)
1 M post-second dose	150	24.31 (18–32)	157	11.95 (9.10–16)	0.49 (0.37–0.66)

hSBA, serum bactericidal assay using human complement; 4CMenB, 4-component meningococcal serogroup B vaccine; MenABCWY, pentavalent meningococcal serogroup A, B, C, W and Y vaccine; N, number of participants; GMT, geometric mean titer; CI, confidence interval; fHbp, factor H binding protein; M, month; NadA, *Neisseria* adhesin; NHBA, Neisserial heparin binding antigen; PorA, porin A.

Bolded values indicate that the lower limit of the 95% CI for the GMT ratio (MenABCWY-0-2 group versus 4CMenB-0-2 group) was >0.5 (non-inferiority criterion).

**Figure 2.**
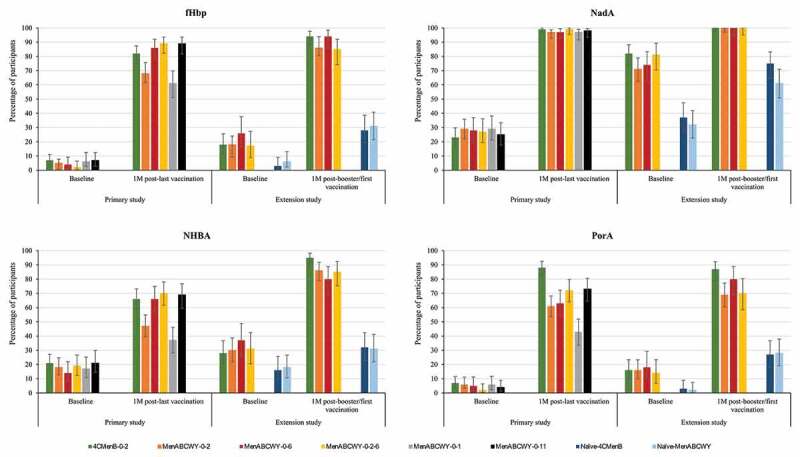
Percentages of participants with hSBA titers ≥LLOQ against antigen-specific MenB test strains, pre- and post-meningococcal vaccination in the primary study and pre- and post-booster (previously vaccinated participants) or post-first meningococcal vaccination (naïve participants) in the extension study (full analysis set).

Across all schedules, administration of the MenABCWY vaccine induced an immune response with increased antibody titers 1 month after the last meningococcal vaccination compared to pre-vaccination titers. The highest immune responses in MenABCWY groups were observed for the 0–6 M, 0-2-6 M and 0–11 M schedules. The percentages of participants with hSBA titers ≥LLOQ were comparable between these 3 groups (with overlapping 95% CIs). Point estimates were similar across these groups for fHbp (86–89%), NadA (97–99%) and NHBA (66–70%), but tended to be slightly lower in the MenABCWY-0-6 group (63%) than the MenABWCY-0-2-6 (72%) and MenABCWY-0-11 (73%) groups for PorA. For the MenABCWY-0-2 group, point estimates were lower with 68%, 97%, 47% and 61% for fHbp, NadA, NHBA and PorA, respectively. Point estimates for the 4CMenB-0-2 group were 82%, 99%, 66% and 88% for fHbp, NadA, NHBA and PorA, respectively (Figure 2 and Table S1a). The same trend was observed for antibody GMTs for all antigens (Table S1b). For the 3 groups with the highest immune response, the percentages of participants with a 4-fold rise in hSBA titers from pre-vaccination levels were higher for fHbp (ranging from 48% to 51%) and NadA (ranging from 93% to 95%) than for PorA (ranging from 24% to 33%) and NHBA (ranging from 19% to 22%) (Figure S1).

hSBA, serum bactericidal assay using human complement; LLOQ, lower limit of quantitation (8.0 for fHbp, 8.6 for NadA, 8.9 for NHBA and 8.2 for PorA); MenB, meningococcal serogroup B; fHbp, factor H binding protein; M, month; NadA, *Neisseria* adhesin; NHBA, Neisserial heparin binding antigen; PorA, porin A; 4CMenB, 4-component meningococcal serogroup B vaccine; MenABCWY, pentavalent meningococcal serogroup A, B, C, W and Y vaccine;.Error bars represent 95% confidence intervals.

**Figure 3.**
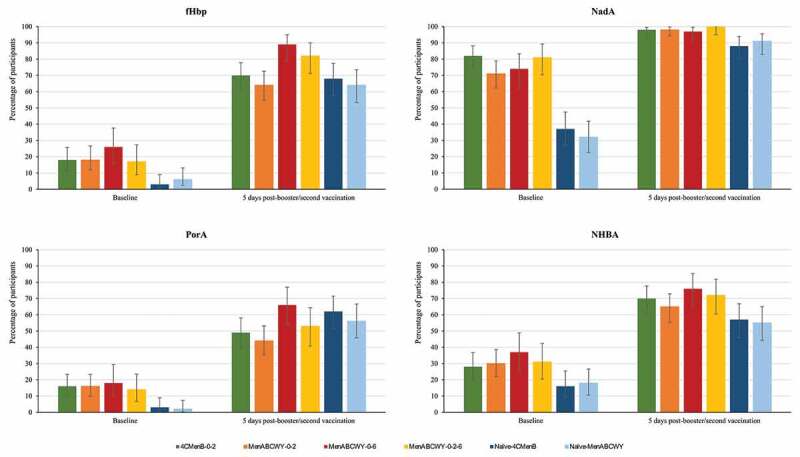
Percentages of participants with hSBA titers ≥LLOQ against antigen-specific MenB test strains, pre-vaccination and 5 days after booster/second vaccination in the extension study (full analysis set).

MenB, meningococcal serogroup B; hSBA, serum bactericidal assay using human complement; LLOQ, lower limit of quantitation (8.0 for fHbp, 8.6 for NadA, 8.9 for NHBA and 8.2 for PorA); 4CMenB, 4-component meningococcal serogroup B vaccine; MenABCWY, pentavalent meningococcal serogroup A, B, C, W and Y vaccine; fHbp, factor H binding protein; NadA, *Neisseria* adhesin; NHBA, Neisserial heparin binding antigen; PorA, porin A.Error bars represent 95% confidence intervals.

In the extension study, the percentages of participants with hSBA titers ≥LLOQ and antibody GMTs were higher at 5 days after booster/second vaccination compared to baseline levels in this study, for all MenB antigens (Figure 3 and Tables S1a and S1b). MenB antibody levels were comparable in the 4CMenB-0-2, MenABCWY-0-2, MenABCWY-0-6 and MenABCWY-0-2-6 groups at 24 months after the last primary vaccination and at 1 month post-booster dose. A higher immune response was observed after a booster dose of either 4CMenB or MenABCWY compared to a first dose of either vaccine in naïve participants, for all MenB antigens. Indeed, one month post-booster/first dose, 85–94% versus 28–31% (fHbp), 100% versus 61–75% (NadA), 80–95% versus 31–32% (NHBA), and 69–87% versus 27–28% of participants had hSBA titers ≥LLOQ in the follow-on versus age-matched naïve groups. (Figure 2 and Table S1a). Antibody GMTs observed at baseline had declined from 1 month post-primary vaccination in follow-on groups, but tended to be higher than pre-primary vaccination and were overall higher than baseline values in vaccine-naïve participants. At 1 month post-booster/first dose, antibody GMTs in follow-on groups were higher than in naïve groups, for all MenB antigens (Table S1b). The percentages of participants witha 4-fold rise in hSBA titers in the extension study ranged from 24% to 66% for fHbp and from 73% to 97% for NadA, from 14% to 40% for PorA and from 15% to 52% for NHBA (Figure S1).

#### Immune response to MenACWY strains

**Figure 4.**
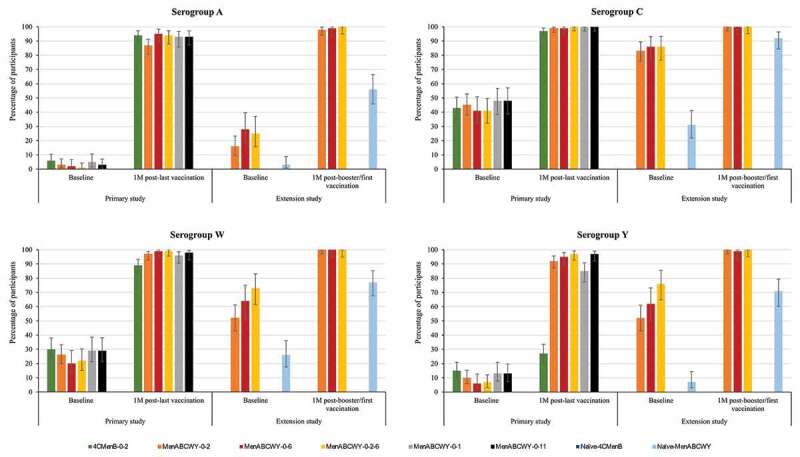
Percentages of participants with hSBA titers ≥LLOQ against meningococcal serogroups A, C, W and Y, pre- and post-meningococcal vaccination in the primary and extension study (full analysis set).

Across all schedules, administration of the MenABCWY vaccine induced an immune response with increased antibody titers 1 month after the last meningococcal vaccination compared to pre-vaccination titers. Overall, the percentage of participants with hSBA titers ≥LLOQ was comparable between all groups receiving the MenABCWY vaccine. Point estimates for all MenABCWY groups ranged from 87%–95% for MenA, 99%–100% for MenC, 96%–99% for MenW and 85%–97% for MenY. For the 4CMenB group, the point estimates were 94%, 97%, 89% and 27% for MenA, MenC, MenW and MenY, respectively (Figure 4 and Table S2a).

hSBA, serum bactericidal assay using human complement; LLOQ, lower limit of quantitation (22.7 for serogroup A, 5.2 for serogroup C, 39.6 for serogroup W and 14.7 for serogroup Y); 4CMenB, 4-component meningococcal serogroup B vaccine; MenABCWY, pentavalent meningococcal serogroup A, B, C, W and Y vaccine; M, month.

Across all groups receiving the MenABCWY vaccine, antibody GMTs increased from pre-vaccination to 1 month after the last meningococcal vaccination in the primary study for each serogroup (Table S2b). Similar to vaccination with MenABCWY, antibody GMTs increased from pre-vaccination to 1 month after 4CMenB vaccination in the primary study for serogroups A, C and W. For serogroup Y, although CIs did not overlap, the observed increase was minimal (Table 3 and Table S2b). The percentages of participants with a 4-fold rise in hSBA titers from pre-vaccination levels for all MenABCWY groups ranged from 42% to 78% for MenA and from 87% to 98% for MenC, from 42% to 89% for MenW and from 58% to 89% for MenY. For the 4CMenB group, these percentages were 56%, 49%, 43% and 4% for MenA, MenC, MenW and MenY, respectively (Figure S2).

**Table 3.** Summary of immune responses against meningococcal serogroups A, C, W and Y in the primary and extension studies in selected groups (adjusted hSBA geometric mean titers)

		Geometric mean titers							
		Serogroup A		Serogroup C		Serogroup W		Serogroup Y	
		4CMenB 0–2	MenABCWY 0–2	4CMenB 0–2	MenABCWY 0–2	4CMenB 0–2	MenABCWY 0–2	4CMenB 0–2	MenABCWY 0-2
Primary study	Baseline	1.37 (1.18–1.59)	1.17 (1.02–1.36)	3.46 (2.76–4.33)	3.38 (2.71–4.23)	6.43 (4.46–9.27)	4.75 (3.35–6.73)	1.72 (1.41–2.10)	1.40 (1.14–1.71)
	1M^a^	106.70 (82–139)	68.85 (53–89)	41.25 (32–52)	184.05 (145–234)	138.43 (111–173)	214.70 (173–266)	3.12 (2.34–4.17)	90.61 (68–121)
Extension study	Baseline	-	2.31 (1.75–3.04)	-	18 (14–23)	-	38 (28–51)	-	9.11 (6.77–12)
	1M^b^	-	270 (222–327)	-	628 (523–753)	-	1345 (1142–1584)	-	623 (517–750)

hSBA, serum bactericidal assay using human complement; 4CMenB, 4-component meningococcal serogroup B vaccine; MenABCWY, pentavalent meningococcal serogroup A, B, C, W and Y vaccine; M, month.

^a^After the last meningococcal vaccination.

^b^After booster/second vaccination.

The lower limit of quantitation was 22.7 for serogroup A, 5.2 for serogroup C, 39.6 for serogroup W and 14.7 for serogroup Y.

In the extension study, antibody levels for all serogroups were comparable in the MenABCWY-0-2, MenABCWY-0-6 and MenABCWY-0-2-6 groups at 24 months after the last primary vaccination and at 1 month post-booster dose. The percentages of participants with hSBA titers ≥LLOQ 1 month after booster/first dose were 98%–100% versus 56% for MenA, 100% versus 92% for MenC, 100% versus 77% for MenW and 99%–100% versus 71% for MenY, for the follow-on versus age-matched naïve groups (Figure 4 and Table S2a). Antibody GMTs observed at baseline had declined from 1 month after the primary vaccination in follow-on groups but were higher than pre-primary vaccination values and baseline values in vaccine-naïve participants. At 1 month post-booster/first dose, antibody GMTs in follow-on groups were higher than in the vaccine-naïve group, across all serogroups (Table S2b). The percentages of participants with a 4-fold rise in hSBA titers in the extension study ranged from 46% to 88% for MenA and from 86% to 94% for MenC, from 71% to 93% for MenW and from 83% to 94% for MenY. (Figure S2).

### Safety

In the primary study, the most frequently reported solicited local AE after any meningococcal vaccination was pain, reported by 81–97% of participants, which was much higher than after any dose of HepA (26–53%). Fatigue, reported by 69–76% of participants, and headache reported by 63–72% of participants were the most frequently reported solicited systemic AEs after any vaccination. Table S3 gives an overview of solicited local and systemic AEs by vaccination. No increase of local or general solicited AEs was observed, even after 3 MenABCWY doses. Unsolicited AEs were reported with similar frequencies in all groups and of the 35 SAEs reported, only 2 were considered as related to study vaccinations (1 seizure in the MenABCWY-0-2 group and 1 connective tissue disorder in the MenABCWY-0-1 group) (Table S4).

In the extension study, pain was the most frequently reported solicited local AE, by 83–95% of participants across all groups and headache and fatigue were the most frequently reported general solicited AEs. For the naïve groups, no increase of local or general solicited AEs was observed after the second dose (Table S5). Unsolicited AEs were reported by 19–49% of participants in all groups. Overall, 3 SAEs (none related to vaccination) were reported (Table S4).

## Discussion

A combined vaccine with MenACWY polysaccharide protein conjugates and MenB proteins is clearly desirable. This investigational MenABCWY vaccine was made by reconstituting the lyophilized MenACWY vaccine with the liquid 4CMenB vaccine. These studies assessed the immunogenicity and safety of the investigational MenABCWY vaccine when administered according to various schedules to adolescents 10–18 years of age and evaluated persistence and booster response of antibodies against MenB test strains, included in the composition of MenABCWY, as well as the MenACWY serogroup response.

While a combined vaccine with MenACWY polysaccharide protein conjugates combined with MenB proteins is clearly desirable, non-inferiority of MenABCWY versus 4CMenB could not be formally demonstrated as defined in the study protocol for the 0–2 M schedule. However, in the primary study, at 1 month after the last meningococcal vaccination, the percentage of participants with hSBA titers ≥LLOQ increased substantially compared with baseline levels for all antigen-specific MenB strains. The responses were lowest for Por A, which is the least important of the 4 components.

Additionally, while hSBA titers in the primary study were lower in the MenABCWY-0-2 group compared to the 4CMenB-0-2 group 1 month after the second vaccination for all MenB antigens, this difference was less pronounced in MenABCWY-recipients when doses were administered more than 2 months apart. These results indicate that a longer interval of at least 6 months between doses is preferable for MenABCWY investigational vaccines.

Furthermore, at 24 months following last vaccination in the primary study, the percentage of participants with hSBA titers ≥LLOQ for each MenB antigen was similar across schedules and vaccines assessed and consistently higher than in a naïve population across indicator MenB strains, indicating that the minor differences between schedules in the primary study may not be clinically significant. In a different study assessing antibody persistence in adolescents and young adults, antibody levels above pre-vaccination values were maintained up to 4 years post-vaccination with MenABCWY for some MenB strains.^34^

Following vaccination with a booster dose in the follow-on groups or a first dose of either 4CMenB or MenABCWY in the naïve participants, higher immune responses for all MenB antigens were observed in the follow-on groups, demonstrating an anamnestic response in previously primed participants, in line with other reports.^34,36,37^

Despite the overall lower response observed for the 0–2 M schedule for MenABCWY versus 4CMenB, the difference was less pronounced for other MenABCWY schedules and comparable antibody persistence and anamnestic response were observed for all 4 MenB antigens across all groups. In addition, a study assessing the effectiveness of 2 MenABCWY doses (with a 0–2 month schedule) against a panel of 110 invasive MenB strains circulating in the US showed that 67% (95% CI: 65–69) of strains were killed 1 month after the second dose when using sera collected from vaccinated healthy adolescents aged 10–18 years.^39^ This approach demonstrates broad protection conferred by MenABCWY against MenB strains and indicates that the assessment of immune responses against antigen-specific laboratory test strains does not fully account for the interplay of humoral immune responses elicited by vaccination. Previous studies have shown that synergistic effects occur between antibodies directed against multiple epitopes on the same or different antigens contained in 4CMenB. Therefore, immune responses induced by antigen combinations may still lead to bactericidal killing even in the absence of expression levels of a single antigen sufficient to induce bactericidal killing.^44,45^

Early immune responses were observed after a single dose of MenABCWY, with 27–75% of participants having hSBA titers ≥LLOQ across all MenB test strains at 1 month after the first vaccination in previously naïve participants. Moreover, immune responses against all 4 antigen-specific MenB strains increased rapidly following either the booster dose in primed participants or a 2-dose vaccination series in naïve participants, as shown by immunogenicity data at 5 days after the booster/second dose vaccination. Considering the fast incubation period of *N. meningitidis*, ranging from 2 to 10 days, our data indicate that the completion of the 0–1 M vaccination schedule with MenABCWY in previously unexposed individuals and administration of a MenABCWY booster dose in previously-primed individuals would ensure a relatively fast response in case of an outbreak or epidemic caused by MenB.^1^ Additional benefits of a MenABCWY pentavalent formulation may go beyond protection against meningococcal serogroups as there is emerging evidence that 4CMenB may, to some extent, provide protection against other genetically-related species, like *Neisseria gonorrhoeae*.^46,47^

As shown in the primary study, 4CMenB vaccination has an impact on 3 non-B strains (MenA, MenC and MenW), with a smaller impact on MenY, indicating cross-reactive immunity of 4CMenB against non-B serogroups. This cross-reactive immunity has been previously reported in the literature.^48–52^ For instance, in a panel of 147 MenC, MenW, and MenY clinical isolates collected from three European countries and Brazil, 74.1% and 61.9% of strains were killed in hSBA by sera from 4CMenB-immunized infants^48^ and adolescents,^49^ respectively. However, the use of a combined MenABCWY vaccine would provide protection against five of the most clinically relevant meningococcal serogroups, with the added benefit of lowering the number of injections as compared with separate MenACWY and 4CMenB vaccination and therefore potentially improving compliance among adolescents and young individuals.

Overall, the safety profile of MenABCWY was similar to that of 4CMenB, with comparable tolerability, regardless of the schedule used. No increase in reactogenicity was observed even after 3 MenABCWY doses. These outcomes confirm previous results in adolescents and young adults aged 10–25 years.^33,35^ No new safety concerns were identified following booster vaccination or 2-dose vaccination in naïve participants, consistent with previous reports.^34,37^

Several vaccination schedules were considered in this study to investigate whether a certain flexibility in the timing of doses is possible without impacting the immunogenicity and safety of MenABCWY. Although a larger interval between doses may increase the immune response, a shorter interval (e.g., the 0–1 month schedule) may provide a rapid response in case of an outbreak. The study also had several potential limitations. The primary study was powered for demonstrating non-inferiority of MenABCWY to 4CMenB when administered according to a 0–2 M schedule (commonly used for vaccination against MenB, including in the US), but not for evaluating differences between MenABCWY schedules. Non-inferiority of MenABCWY to the MenACWY-CRM vaccine was not assessed, however, it was demonstrated in a previous study.^33^ In addition, there was a high number of exclusions during month 2 and month 3 in the primary study.

## Conclusions

MenABCWY vaccination was immunogenic against 4 MenB test strains and serogroups A, C, W and Y, offering broad coverage against MenB strains, although the primary immunogenicity objective was not met. All schedules tested were immunogenic, but a longer time interval (6 or 11 months rather than 2 months) between doses of MenABCWY resulted in a better immune response. Following vaccination with MenABCWY in adolescents, antibodies persisted up to 2 years and a booster dose induced an anamnestic response. MenABCWY had a clinically-acceptable safety profile, with no identified safety concerns.

## Supplementary Material

Supplemental Material

## Data sharing statement

The results summary for this study (GSK studies number 205215-NCT02212457 and 205613-NCT02946385) are available on the GSK Clinical Study Register and can be accessed at www.gsk-clinicalstudyregister.com. For interventional studies that evaluate our medicines, anonymized patient-level data will be made available to independent researchers, subject to review by an independent panel, at www.clinicalstudydatarequest.com within 6 months of publication. To protect the privacy of patients and individuals involved in our studies, GSK does not publicly disclose patient-level data.

## Trademark statement

*Menveo, Bexsero* and *Havrix* are trademarks owned by or licensed to the GSK group of companies.

## References

[1] World Health Organization. Meningococcal meningitis. Key facts. 2018 Feb 19 [accessed 2019 Feb]. https://www.who.int/en/news-room/fact-sheets/detail/meningococcal-meningitis.

[2] Centers for Disease Control and Prevention. Enhanced meningococcal disease surveillance report, 2017. 2019 May 31 [accessed 2020 Feb]. https://www.cdc.gov/meningococcal/downloads/NCIRD-EMS-Report-2017.pdf.

[3] European Center for Disease Prevention and Control. Surveillance report. annual epidemiological report for 2016. Invasive meningococcal disease. Stockholm; 2018.

[4] European Center for Disease Prevention and Control. Factsheet about meningococcal disease. 2019 Jan 7 [accessed 2019 Feb]. https://ecdc.europa.eu/en/meningococcal-disease/factsheet.

[5] Lahra MM, Enriquez R. Australian meningococcal surveillance programme annual report, 2016. Commun Dis Intell Q Rep. 2017;41:E369–E382. 29864389 10.33321/cdi.2017.41.46

[6] Cohn AC, MacNeil JR, Harrison LH, Hatcher C, Theodore J, Schmidt M, Pondo T, Arnold KE, Baumbach J, Bennett N, et al. Changes in Neisseria meningitidis disease epidemiology in the United States, 1998-2007: implications for prevention of meningococcal disease. Clin Infect Dis. 2010;50(2):184–91. doi:10.1086/649209. 20001736

[7] World Health Organization. Invasive meningococcal disease - Serogroup distribution, 2018. 2018 Feb 16 [accessed 2019 Feb]. https://www.who.int/emergencies/diseases/meningitis/serogroup-distribution-2018.pdf?ua=1.

[8] Centers for Disease Control and Prevention. Meningococcal disease. Meningococcal disease in other countries. 2019 May 31 [accessed 2020 Feb]. https://www.cdc.gov/meningococcal/global.html.

[9] Villena R, Safadi MAP, Valenzuela MT, Torres JP, Finn A, O’Ryan M. Global epidemiology of serogroup B meningococcal disease and opportunities for prevention with novel recombinant protein vaccines. Hum Vaccin Immunother. 2018;14(5):1042–57. doi:10.1080/21645515.2018.1458175. 29667483 PMC5989912

[10] Australian Government Departement of Health. Meningococcal disease in Australia. 2020 Mar 20 [accessed 2020 Aug 14]. https://www1.health.gov.au/internet/main/publishing.nsf/Content/ohp-meningococcal-W.htm.

[11] Peterson ME, Li Y, Bita A, Moureau A, Nair H, Kyaw MH, Meningococcal Surveillance G, Abad R, Bailey F, Garcia IF, et al. Meningococcal serogroups and surveillance: a systematic review and survey. J Glob Health. 2019;9(1):010409. doi:10.7189/jogh.09.010409. 30603079 PMC6304171

[12] Christensen H, May M, Bowen L, Hickman M, Trotter CL. Meningococcal carriage by age: a systematic review and meta-analysis. Lancet Infect Dis. 2010;10(12):853–61. doi:10.1016/S1473-3099(10)70251-6. 21075057

[13] Gianchecchi E, Piccini G, Torelli A, Rappuoli R, Montomoli E. An unwanted guest: *Neisseria meningitidis* - carriage, risk for invasive disease and the impact of vaccination with insight on Italy incidence. Expert Rev Anti Infect Ther. 2017;15(7):689–701. doi:10.1080/14787210.2017.1333422. 28524748

[14] Pelton SI. The global evolution of meningococcal epidemiology following the introduction of meningococcal vaccines. J Adolesc Health. 2016;59(2 Suppl):S3–S11. doi:10.1016/j.jadohealth.2016.04.012. 27449148

[15] Crum-Cianflone N, Sullivan E. Meningococcal vaccinations. Infect Dis Ther. 2016;5(2):89–112. doi:10.1007/s40121-016-0107-0. 27086142 PMC4929086

[16] Block SL, Shepard J, Garfield H, Xie F, Han L, Dull PM, Smolenov I. Immunogenicity and safety of a 3- and 4-dose vaccination series of a meningococcal ACWY conjugate vaccine in infants: results of a phase 3b, randomized, open-label trial. Pediatr Infect Dis J. 2016;35(2):e48–59. doi:10.1097/INF.0000000000000965. 26479973

[17] Findlow J, Bai X, Findlow H, Newton E, Kaczmarski E, Miller E, Borrow R. Safety and immunogenicity of a four-component meningococcal group B vaccine (4CMenB) and a quadrivalent meningococcal group ACWY conjugate vaccine administered concomitantly in healthy laboratory workers. Vaccine. 2015;33(29):3322–30. doi:10.1016/j.vaccine.2015.05.027. 26025807

[18] Kimura A, Toneatto D, Kleinschmidt A, Wang H, Dull P. Immunogenicity and safety of a multicomponent meningococcal serogroup B vaccine and a quadrivalent meningococcal CRM197 conjugate vaccine against serogroups A, C, W-135, and Y in adults who are at increased risk for occupational exposure to meningococcal isolates. Clin Vaccine Immunol. 2011;18(3):483–86. doi:10.1128/cvi.00304-10. 21177912 PMC3067382

[19] Gasparini R, Tregnaghi M, Keshavan P, Ypma E, Han L, Smolenov I. Safety and immunogenicity of a quadrivalent meningococcal conjugate vaccine and commonly administered vaccines after coadministration. Pediatr Infect Dis J. 2016;35(1):81–93. doi:10.1097/INF.0000000000000930. 26398743

[20] Halperin SA, Diaz-Mitoma F, Dull P, Anemona A, Ceddia F. Safety and immunogenicity of an investigational quadrivalent meningococcal conjugate vaccine after one or two doses given to infants and toddlers. Eur J Clin Microbiol Infect Dis. 2010;29(3):259–67. doi:10.1007/s10096-009-0848-8. 20033465

[21] Nolan TM, Nissen MD, Naz A, Shepard J, Bedell L, Hohenboken M, Odrljin T, Dull PM. Immunogenicity and safety of a CRM-conjugated meningococcal ACWY vaccine administered concomitantly with routine vaccines starting at 2 months of age. Hum Vaccin Immunother. 2014;10(2):280–89. doi:10.4161/hv.27051. 24220326 PMC4185919

[22] Tregnaghi M, Lopez P, Stamboulian D, Grana G, Odrljin T, Bedell L, Dull PM. Immunogenicity and safety of a quadrivalent meningococcal polysaccharide CRM conjugate vaccine in infants and toddlers. Int J Infect Dis. 2014;26:22–30. doi:10.1016/j.ijid.2014.03.1390. 24980467

[23] Cooper B, DeTora L, Stoddard J. Menveo(R)): a novel quadrivalent meningococcal CRM197 conjugate vaccine against serogroups A, C, W-135 and Y. Expert Rev Vaccines. 2011;10(1):21–33. doi:10.1586/erv.10.147. 21162617

[24] Food and Drug Administration. Approval Letter - BEXSERO. 2015 Jan 23. https://www.fda.gov/media/90996/download.

[25] European Medicines Agency. Bexsero. Summary of product characteristics. 2018 Sept 18 [accessed 2019 Mar 6]. https://www.ema.europa.eu/en/documents/product-information/bexsero-epar-product-information_en.pdf.

[26] Parikh SR, Andrews NJ, Beebeejaun K, Campbell H, Ribeiro S, Ward C, White JM, Borrow R, Ramsay ME, Ladhani SN. Effectiveness and impact of a reduced infant schedule of 4CMenB vaccine against group B meningococcal disease in England: a national observational cohort study. Lancet. 2016;388(10061):2775–82. doi:10.1016/S0140-6736(16)31921-3. 28100432

[27] Prymula R, Esposito S, Zuccotti GV, Xie F, Toneatto D, Kohl I, Dull PM. A phase 2 randomized controlled trial of a multicomponent meningococcal serogroup B vaccine (I). Hum Vaccin Immunother. 2014;10(7):1993–2004. doi:10.4161/hv.28666. 25424809 PMC4186040

[28] Esposito S, Prymula R, Zuccotti GV, Xie F, Barone M, Dull PM, Toneatto D. A phase 2 randomized controlled trial of a multicomponent meningococcal serogroup B vaccine, 4CMenB, in infants (II). Hum Vaccin Immunother. 2014;10(7):2005–14. doi:10.4161/hv.29218. 25424810 PMC4186018

[29] Vesikari T, Esposito S, Prymula R, Ypma E, Kohl I, Toneatto D, Dull P, Kimura A; group EUMBIVS. Immunogenicity and safety of an investigational multicomponent, recombinant, meningococcal serogroup B vaccine (4CMenB) administered concomitantly with routine infant and child vaccinations: results of two randomised trials. Lancet. 2013;381(9869):825–35. doi:10.1016/S0140-6736(12)61961-8. 23324563

[30] Gossger N, Snape MD, Yu LM, Finn A, Bona G, Esposito S, Principi N, Diez-Domingo J, Sokal E, Becker B, et al. Immunogenicity and tolerability of recombinant serogroup B meningococcal vaccine administered with or without routine infant vaccinations according to different immunization schedules: a randomized controlled trial. JAMA. 2012;307(6):573–82. doi:10.1001/jama.2012.85. 22318278

[31] Findlow J, Borrow R, Snape MD, Dawson T, Holland A, John TM, Evans A, Telford KL, Ypma E, Toneatto D, et al. Multicenter, open-label, randomized phase II controlled trial of an investigational recombinant Meningococcal serogroup B vaccine with and without outer membrane vesicles, administered in infancy. Clin Infect Dis. 2010;51(10):1127–37. doi:10.1086/656741. 20954968

[32] Ladhani SN, Ramsay M, Borrow R, Riordan A, Watson JM, Pollard AJ. Enter B and W: two new meningococcal vaccine programmes launched. Arch Dis Child. 2016;101(1):91–95. doi:10.1136/archdischild-2015-308928. 26672098 PMC4717420

[33] Block SL, Szenborn L, Daly W, Jackowska T, D’Agostino D, Han L, Dull PM, Smolenov I. A comparative evaluation of two investigational meningococcal ABCWY vaccine formulations: results of a phase 2 randomized, controlled trial. Vaccine. 2015;33(21):2500–10. doi:10.1016/j.vaccine.2015.03.001. 25795256

[34] Saez-Llorens X, Beltran-Rodriguez J, Novoa Pizarro JM, Mensi I, Keshavan P, Toneatto D. Four-year antibody persistence and response to a booster dose of a pentavalent MenABCWY vaccine administered to healthy adolescents and young adults. Hum Vaccin Immunother. 2018;14(5):1161–74. doi:10.1080/21645515.2018.1457595. 29601256 PMC5989907

[35] Saez-Llorens X, Aguilera Vaca DC, Abarca K, Maho E, Grana MG, Heijnen E, Smolenov I, Dull PM. Immunogenicity and safety of investigational vaccine formulations against meningococcal serogroups A, B, C, W, and Y in healthy adolescents. Hum Vaccin Immunother. 2015;11(6):1507–17. doi:10.1080/21645515.2015.1029686. 25969894 PMC4514249

[36] Saez-Llorens X, Aguilera Vaca DC, Abarca K, Maho E, Han L, Smolenov I, Dull P. Persistence of meningococcal antibodies and response to a third dose after a two-dose vaccination series with Investigational MenABCWY vaccine formulations in adolescents. Pediatr Infect Dis J. 2015;34(10):e264–278. doi:10.1097/INF.0000000000000822. 26135245

[37] Szenborn L, Block SL, Jackowska T, Konior R, D’Agostino D, Smolenov I, Toneatto D, Welsch JA. immune responses to booster vaccination with meningococcal ABCWY vaccine after primary vaccination with either investigational or licensed vaccines: a phase 2 randomized study. Pediatr Infect Dis J. 2018;37(5):475–82. doi:10.1097/INF.0000000000001896. 29329168

[38] Centers for Disease Control and Prevention. Meningococcal vaccination: recommendations of the advisory committee on immunization practices, United States, 2020. MMWR. 2020;69(9):1–41. 10.15585/mmwr.rr6909a1 PMC7527029 33417592

[39] Welsch JA, Senders S, Essink B, Klein T, Smolenov I, Pedotti P, Barbi S, Verma B, Toneatto D. Breadth of coverage against a panel of 110 invasive disease isolates, immunogenicity and safety for 2 and 3 doses of an investigational MenABCWY vaccine in US adolescents - Results from a randomized, controlled, observer-blind phase II study. Vaccine. 2018;36(35):5309–17. doi:10.1016/j.vaccine.2018.07.016. 30061029

[40] World Health Organization. WHO package insert, *Havrix* 1440 Adult/*Havrix* 720 Junior. GDS011/WHO Insert02; 2013 [accessed 2019 Mar 6]. https://www.who.int/immunization_standards/vaccine_quality/pq_267_hepa_GSK_PI_v2.pdf?ua=1.

[41] Mak PA, Santos GF, Masterman KA, Janes J, Wacknov B, Vienken K, Giuliani M, Herman AE, Cooke M, Mbow ML, et al. Development of an automated, high-throughput bactericidal assay that measures cellular respiration as a survival readout for Neisseria meningitidis. Clin Vaccine Immunol. 2011;18(8):1252–60. doi:10.1128/CVI.05028-11. 21715580 PMC3147359

[42] Goldschneider I, Gotschlich EC, Artenstein MS. Human immunity to the meningococcus. I. The role of humoral antibodies. J Exp Med. 1969;129(6):1307–26. doi:10.1084/jem.129.6.1307. 4977280 PMC2138650

[43] Perrett KP, McVernon J, Richmond PC, Marshall H, Nissen M, August A, Percell S, Toneatto D, Nolan T. Immune responses to a recombinant, four-component, meningococcal serogroup B vaccine (4CMenB) in adolescents: a phase III, randomized, multicentre, lot-to-lot consistency study. Vaccine. 2015;33(39):5217–24. doi:10.1016/j.vaccine.2015.06.103. 26232542

[44] Natali EN, Principato S, Ferlicca F, Bianchi F, Fontana LE, Faleri A, Pansegrau W, Surdo PL, Bartolini E, Santini L, et al. Synergic complement-mediated bactericidal activity of monoclonal antibodies with distinct specificity. FASEB J. 2020;34(8):10329–41. doi:10.1096/fj.201902795R. 32725956

[45] Giuliani M, Bartolini E, Galli B, Santini L, Lo Surdo P, Buricchi F, Bruttini M, Benucci B, Pacchiani N, Alleri L, et al. Human protective response induced by meningococcus B vaccine is mediated by the synergy of multiple bactericidal epitopes. Sci Rep. 2018;8(1):3700. doi:10.1038/s41598-018-22057-7. 29487324 PMC5829249

[46] Hadad R, Jacobsson S, Pizza M, Rappuoli R, Fredlund H, Olcen P, Unemo M. Novel meningococcal 4CMenB vaccine antigens - prevalence and polymorphisms of the encoding genes in *Neisseria gonorrhoeae*. APMIS. 2012;120(9):750–60. doi:10.1111/j.1600-0463.2012.02903.x. 22882265

[47] Petousis-Harris H, Paynter J, Morgan J, Saxton P, McArdle B, Goodyear-Smith F, Black S. Effectiveness of a group B outer membrane vesicle meningococcal vaccine against gonorrhoea in New Zealand: a retrospective case-control study. Lancet. 2017;390(10102):1603–10. doi:10.1016/S0140-6736(17)31449-6. 28705462

[48] Biolchi A, De Angelis G, Moschioni M, Tomei S, Brunelli B, Giuliani M, Bambini S, Borrow R, Claus H, Gorla MCO, et al. Multicomponent meningococcal serogroup B vaccination elicits cross-reactive immunity in infants against genetically diverse serogroup C, W and Y invasive disease isolates. Vaccine. 2020;38(47):7542–50. doi:10.1016/j.vaccine.2020.09.050. 33036804

[49] Biolchi A, Tomei S, Brunelli B, Giuliani M, Bambini S, Borrow R, Claus H, Gorla MCO, Hong E, Lemos APS, et al. 4CMenB immunization induces serum bactericidal antibodies against non-serogroup B meningococcal strains in adolescents. Infect Dis Ther. 2021;10(1):307–16. doi:10.1007/s40121-020-00370-x. 33185849 PMC7954916

[50] Fazio C, Biolchi A, Neri A, Tomei S, Vacca P, Ambrosio L, Palmieri A, Mori E, La Gaetana R, Pizza M, et al. Cross-reactivity of 4CMenB vaccine-induced antibodies against meningococci belonging to non-B serogroups in Italy. Hum Vaccin Immunother. 2021;17(7):2225–31. doi:10.1080/21645515.2020.1855951. 33522380 PMC8189125

[51] Hong E, Giuliani MM, Deghmane AE, Comanducci M, Brunelli B, Dull P, Pizza M, Taha MK. Could the multicomponent meningococcal serogroup B vaccine (4CMenB) control *Neisseria meningitidis* capsular group X outbreaks in Africa? Vaccine. 2013;31(7):1113–16. doi:10.1016/j.vaccine.2012.12.022. 23261039

[52] Ladhani SN, Campbell H, Andrews N, Parikh SR, White J, Edelstein M, Clark SA, Lucidarme J, Borrow R, Ramsay ME. First real world evidence of meningococcal group B vaccine, 4CMenB, protection against meningococcal group W disease; prospective enhanced national surveillance, England. Clin Infect Dis. 2020:ciaa1244. doi:10.1093/cid/ciaa1244. 32845996

