# Supplementary material for: Immunogenicity and safety of different schedules of the meningococcal ABCWY vaccine, with assessment of long-term antibody persistence and booster responses – results from two phase 2b randomized trials in adolescents
Source: Hum Vaccin Immunother. 2021 Sep 28;17(11):4689–700. doi: 10.1080/21645515.2021.1968214 (PMC8828153; doi:10.1080/21645515.2021.1968214)
Supplement: Supplemental Material [file KHVI_A_1968214_SM5090.docx]

**Immunogenicity and safety of different schedules of the meningococcal ABCWY vaccine, with assessment of long-term antibody persistence and booster responses – results from two phase 2b randomized trials in adolescents.**

Timo Vesikari^1^, Jerzy Brzostek^2^, Anitta Ahonen^3^, Marita Paassilta^4^, Ewa Majda-Stanislawska^5^, Leszek Szenborn^6^, Miia Virta^3^, Robert Clifford^7^, Teresa Jackowska^8^, Murray Kimmel^9^, Ilaria Bindi^10^, Pavitra Keshavan^10^, Paola Pedotti^11^, Daniela Toneatto^10^

# *^1^Nordic Research Network Oy, Tampere, Finland*

*^2^Health Care Establishment in Debica, Infectious Diseases Outpatient Clinic, Debica, Poland*

*^3^Tampere University, Vaccine Research Center, Finland*

*^4^Tampere University and Tampere University Hospital, Finland*

*^5^Department of Pediatric Infectious Diseases, Medical University of Lodz, Lodz, Poland*

*^6^Department of Pediatric Infectious Diseases, Wroclaw Medical University, Wroclaw, Poland*

*^7^Coastal Pediatric Research, Charleston, South Carolina, United States*

*^8^Department of Pediatrics, The Medical Centre of Postgraduate Education, Warsaw, Poland*

*^9^Optimal Research LLC, Melbourne, Florida, United States*

*^10^GSK, Siena, Italy*

*^11^GSK, Amsterdam, The Netherlands*

**Corresponding author:** Daniela Toneatto, GSK, Via Fiorentina, 1, 53100 Siena, Italy ([daniela.x.toneatto@gsk.com](mailto:daniela.x.toneatto@gsk.com))

# Supplemental material

# Table S1a. Summary of immune responses against MenB test strains in the primary and extension studies (percentages of participants with hSBA titers ≥LLOQs)

|  |  | 4CMenB-0-2 | |  | | MenABCWY-0-2 |  | | MenABCWY-0-6 |  | MenABCWY-0-2-6 | |  | MenABCWY-0-1 | |  | MenABCWY-0-11 | |  | | Naïve-4CMenB | |  | Naïve-MenABCWY |
| --- | --- | --- | --- | --- | --- | --- | --- | --- | --- | --- | --- | --- | --- | --- | --- | --- | --- | --- | --- | --- | --- | --- | --- | --- |
|  |  | N | %  (95%CI) |  | N | %  (95%CI) |  | N | %  (95%CI) |  | N | %  (95%CI) |  | N | %  (95%CI) |  | N | %  (95%CI) |  | N | %  (95%CI) |  | N | %  (95%CI) |
| **fHbp** |  |  |  |  |  |  |  |  |  |  |  |  |  |  |  |  |  |  |  |  |  |  |  |  |
| Baseline in primary study |  | 184 | 7  (3.41‒11.11) |  | 179 | 5  (2.32–9.33) |  | 108 | 4  (1.02–9.21) |  | 134 | 2  (0.46–6.40) |  | 112 | 6  (2.55-12.45) |  | 123 | 7  (2.85–12.41) |  |  |  |  |  |  |
| 1M post-last men. vaccination |  | 184 | 82  (75.75‒87.32) |  | 179 | 68  (60.21-74.39) |  | 108 | 86  (78.13–92.01) |  | 134 | 89  (82.21–93.60) |  | 112 | 61  (51.04–69.81) |  | 123 | 89  (81.64 –93.64) |  |  |  |  |  |  |
| Baseline in extension study |  | 119 | 18  (11.3–25.7) |  | 119 | 18  (12.0–26.6) |  | 70 | 26  (16.0–37.6) |  | 72 | 17  (8.9–27.3) |  |  |  |  |  |  |  | 94 | 3  (0.7–9.0) |  | 96 | 6  (2.3–13.1) |
| 5D post-booster/second vaccination |  | 119 | 70  (60.7‒77.8) |  | 119 | 64  (54.6‒72.5) |  | 70 | 89  (78.7‒94.9) |  | 72 | 82  (71.1‒90.0) |  |  |  |  |  |  |  | 94 | 68  (57.7‒77.3) |  | 94 | 64  (53.3‒73.5) |
| 1M post-booster/first vaccination |  | 119 | 94  (88.3–97.6) |  | 119 | 86  (78.1‒91.5) |  | 70 | 94  (86.0–98.4) |  | 72 | 85  (74.3–92.1) |  |  |  |  |  |  |  | 92 | 28  (19.4–38.6) |  | 95 | 31  (21.5–40.8) |
| **NadA** |  |  |  |  |  |  |  |  |  |  |  |  |  |  |  |  |  |  |  |  |  |  |  |  |
| Baseline in primary study |  | 186 | 23  (17.26‒29.85) |  | 178 | 29  (22.14–35.89) |  | 112 | 28  (19.64–36.93) |  | 121 | 27  (19.57–36.12) |  | 120 | 29  (21.23–38.16) |  | 128 | 25  (17.77– 33.42) |  |  |  |  |  |  |
| 1M post-last men. vaccination |  | 186 | 99  (97.04‒99.99) |  | 178 | 97  (92.81–98.75) |  | 112 | 97  (92.37–99.44) |  | 121 | 99  (95.48–99.98) |  | 120 | 97  (91.69–99.08) |  | 128 | 98  (93.30 – 99.51) |  |  |  |  |  |  |
| Baseline in extension study |  | 121 | 82  (73.8–88.2) |  | 125 | 71  (62.4–78.9) |  | 72 | 74  (61.9–83.3) |  | 74 | 81  (70.3–89.3) |  |  |  |  |  |  |  | 95 | 37  (27.2–47.4) |  | 98 | 32  (22.6–41.8) |
| 5D post-booster/second vaccination |  | 121 | 98  (92.9‒99.5) |  | 125 | 98  (94.3‒99.81) |  | 72 | 97  (90.3‒99.66) |  | 74 | 100  (95.1‒100.0) |  |  |  |  |  |  |  | 94 | 88  (80.0‒94.0) |  | 96 | 91  (82.9‒95.6) |
| 1M post-booster/first vaccination |  | 121 | 100  (97.0–100) |  | 125 | 100  (97.1–100) |  | 72 | 100  (95.0–100) |  | 74 | 100  (95.1–100) |  |  |  |  |  |  |  | 95 | 75  (64.8–83.1) |  | 98 | 61  (50.8–70.9) |
| **PorA** |  |  |  |  |  |  |  |  |  |  |  |  |  |  |  |  |  |  |  |  |  |  |  |  |
| Baseline in primary study |  | 188 | 7  (3.73‒11.53) |  | 185 | 6  (3.40–11.06) |  | 112 | 5  (1.99–11.30) |  | 134 | 2  (0.46–6.40) |  | 120 | 6  (2.38–11.65) |  | 126 | 4  (1.30 – 9.02) |  |  |  |  |  |  |
| 1M post-last men. vaccination |  | 188 | 88  (82.82‒92.52) |  | 185 | 61  (53.65–68.15) |  | 112 | 63  (53.76–72.29) |  | 134 | 72  (64.00–79.76) |  | 120 | 43  (33.53–51.85) |  | 126 | 73  (64.38 – 80.53) |  |  |  |  |  |  |
| Baseline in extension study |  | 121 | 16  (9.7–23.4) |  | 127 | 16  (9.9–23.3) |  | 71 | 18  (10.1–29.3) |  | 74 | 14  (6.7–23.5) |  |  |  |  |  |  |  | 94 | 3  (0.7–9.0) |  | 97 | 2  (0.25–7.3) |
| 5D post-booster/second vaccination |  | 121 | 49  (39.6‒58.0) |  | 127 | 44  (35.3‒53.2) |  | 71 | 66  (54.0‒77.0) |  | 74 | 53  (40.7‒64.4) |  |  |  |  |  |  |  | 94 | 62  (51.1‒71.5) |  | 94 | 56  (45.8‒66.6) |
| 1M post-booster/first vaccination |  | 121 | 87  (79.4–92.2) |  | 127 | 69  (60.5–77.2) |  | 71 | 80  (69.1–88.8) |  | 74 | 70  (58.5–80.3) |  |  |  |  |  |  |  | 94 | 27  (18.0–36.7) |  | 97 | 28  (19.2–37.9) |
| **NHBA** |  |  |  |  |  |  |  |  |  |  |  |  |  |  |  |  |  |  |  |  |  |  |  |  |
| Baseline in primary study |  | 188 | 21  (15.19‒27.25) |  | 176 | 18  (12.78‒24.69) |  | 113 | 14  (8.32–21.97) |  | 128 | 19  (12.40–26.60) |  | 117 | 17  (10.77–25.16) |  | 121 | 21  (14.54 – 29.88) |  |  |  |  |  |  |
| 1M post-last men. vaccination |  | 188 | 66  (59.26‒73.19) |  | 176 | 47  (39.60–54.81) |  | 113 | 66  (56.88–74.99) |  | 128 | 70  (61.60–78.06) |  | 117 | 37  (28.03–46.16) |  | 121 | 69  (59.53-76.73) |  |  |  |  |  |  |
| Baseline in extension study |  | 122 | 28  (20.1–36.7) |  | 124 | 30  (22.0–38.7) |  | 71 | 37  (25.5–48.9) |  | 75 | 31  (20.5–42.4) |  |  |  |  |  |  |  | 92 | 16  (9.4–25.5) |  | 97 | 18  (10.6–26.6) |
| 5D post-booster/second vaccination |  | 122 | 70  (60.7–77.7) vaccination77.7) |  | 124 | 65  (55.4–72.9) |  | 71 | 76  (64.5–85.4) |  | 75 | 72  (60.4–81.8) |  |  |  |  |  |  |  | 92 | 57  (45.8–66.8) |  | 95 | 55  (44.2–65.0) |
| 1M post-booster/first vaccination |  | 122 | 95  (89.6–98.2) |  | 124 | 86  (79.0–91.8) |  | 71 | 80  (69.1–88.8) |  | 75 | 85  (75.3–92.4) |  |  |  |  |  |  |  | 94 | 32  (22.7–42.3) |  | 97 | 31  (21.9–41.1) |

MenB, meningococcal serogroup B; hSBA, serum bactericidal assay using human complement; LLOQ, lower limit of quantitation (8.0 for fHbp, 8.6 for NadA, 8.2 for PorA and 8.9 for NHBA); 4CMenB, 4-component meningococcal serogroup B vaccine; MenABCWY, pentavalent meningococcal serogroup A, B, C, W and Y vaccine; N, number of participants; CI, confidence interval; fHbp, factor H binding protein; M, month; D, days; NadA, Neisseria adhesin; NHBA, Neisserial heparin binding antigen; PorA, porin A.

**Table S1b.** Summary of immune responses against MenB test strains in the primary and extension studies (adjusted hSBA geometric mean titers)

|  |  | Geometric mean titers  (95% CIs) | | | | | | | | | | | | | | |
| --- | --- | --- | --- | --- | --- | --- | --- | --- | --- | --- | --- | --- | --- | --- | --- | --- |
|  |  | 4CMenB-0-2 |  | MenABCWY-0-2 |  | MenABCWY-0-6 |  | MenABCWY-0-2-6 |  | MenABCWY-0-1 |  | MenABCWY-0-11 |  | Naïve-4CMenB |  | Naïve-MenABCWY |
| **fHbp** |  |  |  |  |  |  |  |  |  |  |  |  |  |  |  |  |
| Baseline in primary study |  | 1.37  (1.21–1.56) |  | 1.22  (1.08–1.39) |  | 1.21  (1.04– 1.41) |  | 1.17  (1.02–1.35) |  | 1.22  (1.04–1.42) |  | 1.34  (1.15–1.55) |  |  |  |  |
| 1M post-last men. vaccination |  | 18.29  (15–23) |  | 12.85  (10–16) |  | 27.47  (22– 35) |  | 28.56  (23–35) |  | 9.23  (7.21–12) |  | 29.01  (23–36) |  |  |  |  |
| Baseline in extension study |  | 2.04  (1.67–2.49) |  | 2.08  (1.70–2.54) |  | 2.62  (2.02–3.41) |  | 2.04  (1.58–2.64) |  |  |  |  |  | 1.17  (1.03–1.34) |  | 1.28  (1.12–1.46) |
| 5D post-booster/second vaccination |  | 14  (11–19) |  | 13  (9.77–18) |  | 40  (27–58) |  | 21  (14–31) |  |  |  |  |  | 12  (8.41–16) |  | 10  (7.48–14) |
| 1M post-booster/first vaccination |  | 49  (38–63) |  | 30  (24–39) |  | 61  (44–84) |  | 30  (22–42) |  |  |  |  |  | 3.59  (2.55–5.04) |  | 3.26  (2.33–4.56) |
| **NadA** |  |  |  |  |  |  |  |  |  |  |  |  |  |  |  |  |
| Baseline in primary study |  | 2.32  (1.82–2.95) |  | 2.84  (2.23–3.62) |  | 2.39  (1.78– 3.21) |  | 2.57  (1.94–3.40) |  | 2.88  (2.16–3.83) |  | 2.78  (2.11–3.68) |  |  |  |  |
| 1M post-last men. vaccination |  | 263.56  (211–329) |  | 169.65  (136–212) |  | 264.85  (207– 340) |  | 306.14  (242–388) |  | 124.75  (92–169) |  | 264.32  (205–341) |  |  |  |  |
| Baseline in extension study |  | 20  (15–26) |  | 13  (10–17) |  | 16  (11–23) |  | 19  (14–27) |  |  |  |  |  | 4.43  (3.22–6.09) |  | 3.09  (2.26–4.23) |
| 5D post-booster/second vaccination |  | 341  (259–448) |  | 376  (287–493) |  | 401  (281–571) |  | 486  (343–690) |  |  |  |  |  | 85  (63–116) |  | 70  (51–95) |
| 1M post-booster/first vaccination |  | 822  (692–975) |  | 587  (496–695) |  | 550  (440–687) |  | 524  (421–652) |  |  |  |  |  | 18  (13–26) |  | 10  (7.42–15) |
| **PorA** |  |  |  |  |  |  |  |  |  |  |  |  |  |  |  |  |
| Baseline in primary study |  | 1.22  (1.08–1.38) |  | 1.22  (1.08–1.38) |  | 1.13  (0.97– 1.31) |  | 1.06  (0.93–1.22) |  | 1.21  (1.04–1.40) |  | 1.12  (0.97–1.29) |  |  |  |  |
| 1M post-last men. vaccination |  | 27.17  (22–34) |  | 11.46  (9.28–14) |  | 14.30  (11– 18) |  | 18.24  (14–23) |  | 6.50  (4.98–8.48) |  | 17.08  (14–21) |  |  |  |  |
| Baseline in extension study |  | 1.72  (1.41–2.09) |  | 1.78  (1.47–2.16) |  | 2.17  (1.68–2.80) |  | 1.66  (1.30–2.14) |  |  |  |  |  | 1.15  (1.04–1.28) |  | 1.06  (0.96–1.18) |
| 5D post-booster/second vaccination |  | 6.39  (4.83–8.45) |  | 6.02  (4.58–7.92) |  | 12  (8.56–18) |  | 7.63  (5.33–11) |  |  |  |  |  | 9.38  (6.83–13) |  | 8.95  (6.52–12) |
| 1M post-booster/first vaccination |  | 27  (21–34) |  | 17  (13–21) |  | 17  (12–23) |  | 14  (10–19) |  |  |  |  |  | 3.18  (2.20–4.59) |  | 3.25  (2.26–4.68) |
| **NHBA** |  |  |  |  |  |  |  |  |  |  |  |  |  |  |  |  |
| Baseline in primary study |  | 2.26  (1.81–2.82) |  | 2.07  (1.65–2.59) |  | 2.06  (1.57–2.71) |  | 1.83  (1.42–2.36) |  | 2.01  (1.53–2.63) |  | 2.59  (1.99–3.37) |  |  |  |  |
| 1M post-last men. vaccination |  | 13.91  (12–17) |  | 8.82  (7.29–11) |  | 15.70  (13–20) |  | 19.18  (16–24) |  | 6.73  (5.44–8.34) |  | 15.68  (13–19) |  |  |  |  |
| Baseline in extension study |  | 4.24  (3.30–5.45) |  | 3.97  (3.10–5.09) |  | 5.30  (3.81–7.35) |  | 4.22  (3.07–5.80) |  |  |  |  |  | 2.37  (1.88–2.99) |  | 2.19  (1.75–2.75) |
| 5D post-booster/second vaccination |  | 17  (13–21) |  | 14  (11–18) |  | 16  (12–23) |  | 17  (12–24) |  |  |  |  |  | 11  (8.54–15) |  | 8.68  (6.52–12) |
| 1M post-booster/first vaccination |  | 59  (47–72) |  | 32  (26–40) |  | 22  (16–28) |  | 26  (20–34) |  |  |  |  |  | 4.90  (3.52–6.82) |  | 4.64  (3.35–6.42) |

MenB, meningococcal serogroup B; hSBA, serum bactericidal assay using human complement; 4CMenB, 4-component meningococcal serogroup B vaccine; MenABCWY, pentavalent meningococcal serogroup A, B, C, W and Y vaccine; fHbp, factor H binding protein; M, month; D, days; NadA*, Neisseria* adhesin; PorA, porin A; NHBA, Neisserial heparin binding antigen.

The lower limit of quantitation was 8.0 for fHbp, 8.6 for NadA, 8.2 for PorA and 8.9 for NHBA.

**Table S2a.** Summary of immune responses against meningococcal serogroups A, C, W and Y in the primary and extension studies (percentages of participants with hSBA titers ≥LLOQs)

|  |  | 4CMenB-0-2 | |  | MenABCWY-0-2 | | |  | MenABCWY-0-6 | |  | MenABCWY-0-2-6 | |  | MenABCWY-0-1 | |  | MenABCWY-0-11 | |  | Naïve-MenABCWY | |
| --- | --- | --- | --- | --- | --- | --- | --- | --- | --- | --- | --- | --- | --- | --- | --- | --- | --- | --- | --- | --- | --- | --- |
|  |  | N | %  (95%CI) |  | N |  | %  (95%CI) |  | N | %  (95%CI) |  | N | %  (95%CI) |  | N | %  (95%CI) |  | N | %  (95%CI) |  | N | %  (95%CI) |
| **Serogroup A** |  |  |  |  |  |  |  |  |  |  |  |  |  |  |  |  |  |  |  |  |  |  |
| Baseline in primary study |  | 172 | 6  (2.82–10.43) |  | 178 |  | 3  (1.25–7.19) |  | 105 | 2  (0.23–6.71) |  | 127 | 1  (0.02–4.31) |  | 107 | 5  (1.53–10.57) |  | 120 | 3  (0.52–7.13) |  |  |  |
| 1M post-last men. vaccination |  | 172 | 94  (89.57–97.18) |  | 178 |  | 87  (80.61–91.17) |  | 105 | 95  (89.24–98.44) |  | 127 | 94  (87.97–97.24) |  | 107 | 93  (85.80–96.72) |  | 120 | 93  (87.29–97.08) |  |  |  |
| Baseline in extension study |  |  |  |  | 122 |  | 16  (9.6–23.2) |  | 72 | 28  (17.9–39.6) |  | 71 | 25  (15.8–37.1) |  |  |  |  |  |  |  | 95 | 3  (0.7–9.0) |
| 1M post-booster/ first vaccination |  |  |  |  | 122 |  | 98  (94.2–99.80) |  | 72 | 99  (92.5–99.96) |  | 71 | 100  (94.9–100) |  |  |  |  |  |  |  | 94 | 56  (45.8–66.6) |
| **Serogroup C** |  |  |  |  |  |  |  |  |  |  |  |  |  |  |  |  |  |  |  |  |  |  |
| Baseline in primary study |  | 186 | 43  (35.79–50.46) |  | 185 |  | 45  (38.09–52.87) |  | 114 | 41  (32.09–50.83) |  | 130 | 41  (32.24–49.73) |  | 122 | 48  (38.43–56.78) |  | 123 | 48  (38.88–57.16) |  |  |  |
| 1M post-last men. vaccination |  | 186 | 97  (93.84–99.12) |  | 185 |  | 99  (96.15–99.87) |  | 114 | 99  (95.21–99.98) |  | 130 | 100  (97.20–100) |  | 122 | 100  (97.02–100) |  | 123 | (100  (97.05–100) |  |  |  |
| Baseline in extension study |  |  |  |  | 127 |  | 83  (75.8–89.5) |  | 72 | 86  (75.9–93.1) |  | 74 | 86  (76.5–93.3) |  |  |  |  |  |  |  | 97 | 30  (21.9–41.1) |
| 1M post-booster/first vaccination |  |  |  |  | 127 |  | 100  (97.1–100) |  | 72 | 100  (95.0–100) |  | 74 | 100  (95.1–100) |  |  |  |  |  |  |  | 97 | 92  (84.4–96.4) |
| **Serogroup W** |  |  |  |  |  |  |  |  |  |  |  |  |  |  |  |  |  |  |  |  |  |  |
| Baseline in primary study |  | 162 | 30  (23.29–37.95) |  | 180 |  | 26  (19.86–33.17) |  | 108 | 20  (13.23–29.20) |  | 123 | 22  (14.99–30.31) |  | 119 | 29  (21.42–38.46) |  | 123 | 29  (21.41–38.15) |  |  |  |
| 1M post-last men. vaccination |  | 162 | 89  (83.01–93.28) |  | 180 |  | 97  (92.89–98.77) |  | 108 | 99  (94.95–99.98) |  | 123 | 99  (95.55–99.98) |  | 119 | 96  (90.47–98.62) |  | 123 | 98  (93.04–99.49) |  |  |  |
| Baseline in extension study |  |  |  |  | 121 |  | 52  (42.8–61.2) |  | 69 | 64  (51.3–75.0) |  | 71 | 73  (61.4–83.1) |  |  |  |  |  |  |  | 96 | 26  (17.6–36.0) |
| 1M post-booster/first vaccination |  |  |  |  | 121 |  | 100  (97.0–100) |  | 69 | 100  (94.8–100) |  | 71 | 100  (94.9–100) |  |  |  |  |  |  |  | 97 | 77  (67.7–85.2) |
| **Serogroup Y** |  |  |  |  |  |  |  |  |  |  |  |  |  |  |  |  |  |  |  |  |  |  |
| Baseline in primary study |  | 188 | 15  (10.13–20.80) |  | 180 |  | 10  (6.04–15.34) |  | 110 | 6  (2.60–12.67) |  | 137 | 7  (3.05–12.10) |  | 120 | 13  (7.82–20.75) |  | 127 | 13  (7.38–19.65) |  |  |  |
| 1M post-last men. vaccination |  | 188 | 27  (20.43–33.52) |  | 180 |  | 92  (87.29–95.68) |  | 110 | 95  (88.51–97.97) |  | 137 | 97  (92.69–99.20) |  | 120 | 85  (77.33–90.86) |  | 127 | 97  (92.13–99.14) |  |  |  |
| Baseline in extension study |  |  |  |  | 125 |  | 52  (42.9–61.0) |  | 71 | 62  (49.7–73.2) |  | 72 | 76  (64.9–85.6) |  |  |  |  |  |  |  | 96 | 7  (3.0–14.4) |
| 1M post-booster/first vaccination |  |  |  |  | 125 |  | 100  (97.1–100) |  | 71 | 99  (92.4–99.96) |  | 72 | 100  (95.0–100) |  |  |  |  |  |  |  | 95 | 71  (60.3–79.4) |

hSBA, serum bactericidal assay using human complement; LLOQ, lower limit of quantitation (22.7 for serogroup A, 5.2 for serogroup C, 39.6 for serogroup W and 14.7 for serogroup Y); 4CMenB, 4-component meningococcal serogroup B vaccine; MenABCWY, pentavalent meningococcal serogroup A, B, C, W and Y vaccine; N, number of participants, CI, confidence interval; M, month.

**Table S2b.** Summary of immune responses against meningococcal serogroups A, C, W and Y in the primary and extension studies (adjusted hSBA geometric mean titers)

|  | **Geometric mean titers** | | | | | | | |
| --- | --- | --- | --- | --- | --- | --- | --- | --- |
|  | 4CMenB-0-2 | MenABCWY-0-2 | MenABCWY-0-6 | MenABCWY-0-2-6 | | MenABCWY-0-1 | MenABCWY-0-11 | Naïve-MenABCWY |
| **Serogroup A** |  |  |  | |  |  |  |  |
| Baseline in primary study | 1.37  (1.18–1.59) | 1.17  (1.02–1.36) | 1.08  (0.90–1.30) | | 1.06  (0.90–1.25) | 1.38  (1.15–1.65) | 1.19  (1.00–1.41) |  |
| 1M post-last men. vaccination | 106.70  (82–139) | 68.85  (53–89) | 165.14  (117–233) | | 140.73  (103–193) | 85.49  (59–125) | 168.23  (119–238) |  |
| Baseline in extension study |  | 2.31  (1.75–3.04) | 5.13  (3.59–7.34) | | 4.53  (3.16–6.48) |  |  | 1.21  (1.02–1.44) |
| 1M post-booster/first vaccination |  | 270  (222–327) | 340  (264–437) | | 275  (213–354) |  |  | 18  (12–27) |
| **Serogroup C** |  |  |  | |  |  |  |  |
| Baseline in primary study | 3.46  (2.76–4.33) | 3.38  (2.71–4.23) | 3.36  (2.56–4.40) | | 2.99  (2.32–3.87) | 3.87  (2.96–5.04) | 3.80  (2.92–4.94) |  |
| 1M post-last men. vaccination | 41.25  (32–52) | 184.05  (145–234) | 260.57  (199–342) | | 476.44  (369-615) | 163.28  (118–227) | 303.63  (235–392) |  |
| Baseline in extension study |  | 18  (14–23) | 21  (15–29) | | 29  (21–41) |  |  | 2.88  (2.23–3.71) |
| 1M post-booster/first vaccination |  | 628  (523–753) | 539  (423–686) | | 612  (482–776) |  |  | 50  (34–75) |
| **Serogroup W** |  |  |  | |  |  |  |  |
| Baseline in primary study | 6.43  (4.46–9.27) | 4.75  (3.35–6.73) | 4.29  (2.80–6.58) | | 4.46  (2.97–6.69) | 4.54  (2.98–6.90) | 4.95  (3.30–7.44) |  |
| 1M post-last men. vaccination | 138.43  (111–173) | 214.70  (173–266) | 340.85  (256–453) | | 270.12  (206–354) | 175.57  (128–241) | 478.73  (363–631) |  |
| Baseline in extension study |  | 38  (28–51) | 44  (30–65) | | 51  (35–75) |  |  | 6.33  (4.28–9.37) |
| 1M post-booster/first vaccination |  | 1345  (1142–1584) | 999  (805–1240) | | 1050  (849–1299) |  |  | 132  (94–186) |
| **Serogroup Y** |  |  |  | |  |  |  |  |
| Baseline in primary study | 1.72  (1.41–2.10) | 1.40  (1.14–1.71) | 1.24  (0.97–1.59) | | 1.34  (1.07–1.67) | 1.68  (1.32–2.13) | 1.79  (1.42–2.25) |  |
| 1M post-last men. vaccination | 3.12  (2.34–4.17) | 90.61  (68–121) | 152.73  (111–211) | | 180.33  (135–242) | 63.45  (44–93) | 231.39  (171–313) |  |
| Baseline in extension study |  | 9.11  (6.77–12) | 17  (12–25) | | 31  (21–45) |  |  | 1.53  (1.22–1.92) |
| 1M post-booster/first vaccination |  | 623  (517–750) | 586  (458–750) | | 646  (506–824) |  |  | 34  (21–54) |

hSBA, serum bactericidal assay using human complement; 4CMenB, 4-component meningococcal serogroup B vaccine; MenABCWY, pentavalent meningococcal serogroup A, B, C, W and Y vaccine; M, month.

The lower limit of quantitation was 22,7 for serogroup A, 5.2 for serogroup C, 39.6 for serogroup W and 14.7 for serogroup Y.

# Table S3. Solicited adverse events in the primary study

|  | Number (%) of participants | | | | | |
| --- | --- | --- | --- | --- | --- | --- |
|  | 4CMenB-0-2 | MenABCWY-0-2 | MenABCWY-0-6 | MenABCWY-0-2-6 | MenABCWY-0-1 | MenABCWY-0-11 |
| **Local solicited AEs** | | | | | | |
| M0 | **N=227** | **N=231** | **N=134** | **N=159** | **N=154** | N=151 |
| Erythema | **23 (10%)** | **24 (10%)** | **12 (9%)** | **23 (14%)** | **18 (12%)** | 1 (1%) |
| Severe | **0** | **3 (1%)** | **0** | **2 (1%)** | **1 (1%)** | 0 |
| Induration | **24 (11%)** | **23 (10%)** | **13 (10%)** | **21 (13%)** | **14 (9%)** | 3 (2%) |
| Severe | **0** | **0** | **1 (1%)** | **1 (1%)** | **0** | 0 |
| Pain | **213 (94%)** | **223 (97%)** | **126 (94%)** | **146 (92%)** | **142 (92%)** | 80 (53%) |
| Severe | **9 (4%)** | **13 (6%)** | **10 (7%)** | **6 (4%)** | **2 (1%)** | 0 |
| M1 | N=214 | N=216 | N=128 | N=151 | **N=150** | **N=144** |
| Erythema | 3 (1%) | 5 (2%) | 1 (1%) | 3 (2%) | **9 (6%)** | **17 (12%)** |
| Severe | 0 | 0 | 0 | 1 (1%) | **2 (1%)** | **4 (3%)** |
| Induration | 2 (1%) | 2 (1%) | 0 | 2 (1%) | **12 (8%)** | **12 (8%)** |
| Severe | 0 | 0 | 0 | 0 | **0** | **0** |
| Pain | 32 (15%) | 34 (16%) | 41 (32%) | 51 (34%) | **122 (81%)** | **135 (94%)** |
| Severe | 0 | 1 (<1%) | 0 | 0 | **5 (3%)** | **11 (8%)** |
| M2 | **N=212** | **N=217** | N=125 | **N=150** | N=144 | N=138 |
| Erythema | **33 (16%)** | **29 (13%)** | 2 (2%) | **20 (13%)** | 0 | 0 |
| Severe | **2 (1%)** | **6 (3%)** | 0 | **1 (1%)** | 0 | 0 |
| Induration | **27 (13%)** | **18 (8%)** | 0 | **22 (15%)** | 1 (1%) | 1 (1%) |
| Severe | **2 (1%)** | **1 (<1%)** | 0 | **0** | 0 | 0 |
| Pain | **193 (91%)** | **185 (85%)** | 26 (21%) | **133 (89%)** | 38 (26%) | 16 (12%) |
| Severe | **11 (5%)** | **7 (3%)** | 0 | **5 (3%)** | 0 | 0 |
| M6 | N=206 | N=211 | **N=120** | **N=152** | N=139 | N=136 |
| Erythema | 1 (<1%) | 1 (<1%) | **12 (10%)** | **16 (11%)** | 0 | 0 |
| Severe | 0 | 0 | **1 (1%)** | **5 (3%)** | 0 | 0 |
| Induration | 2 (1%) | 3 (1%) | **10 (8%)** | **14 (9%)** | 0 | 0 |
| Severe | 0 | 0 | **1 (1%)** | **1 (1%)** | 0 | 0 |
| Pain | 79 (38%) | 77 (36%) | **106 (88%)** | **138 (91%)** | 19 (14%) | 44 (32%) |
| Severe | 1 (< 1%) | 0 | **10 (8%)** | **9 (6%)** | 0 | 0 |
| M12 | N=206 | N=206 | N=121 | N=147 | N=140 | **N=132** |
| Erythema | 1 (<1%) | 1 (<1%) | 0 | 0 | 1 (1%) | **11 (8%)** |
| Severe | 0 | 0 | 0 | 0 | 0 | **1 (1%)** |
| Induration | 2 (1%) | 3 (1%) | 2 (2%) | 1 (1%) | 1 (1%) | **16 (12%)** |
| Severe | 0 | 0 | 1 (1%) | 0 | 0 | **1 (1%)** |
| Pain | 83 (40%) | 80 (39%) | 48 (40%) | 64 (44%) | 58 (41%) | **119 (90%)** |
| Severe | 0 | 1 (<1%) | 0 | 1 (1%) | 1 (1%) | **11 (8%)** |
| **General solicited AEs** | | | | | | |
| M0 | **N=227** | **N=231** | **N=134** | **N=159** | **N=154** | N=151 |
| Chills | **45 (20%)** | **36 (16%)** | **24 (18%)** | **40 (25%)** | **41 (27%)** | 27 (18%) |
| Severe | **1 (<1%)** | **0** | **1 (1%)** | **1 (1%)** | **1 (1%)** | 2 (1%) |
| Myalgia | **62 (27%)** | **53 (23%)** | **34 (25%)** | **40 (25%)** | **39 (25%)** | 33 (22%) |
| Severe | **2 (1%)** | **4 (2%)** | **2 (1%)** | **3 (2%)** | **2 (1%)** | 0 |
| Arthralgia | **25 (11%)** | **14 (6%)** | **17 (13%)** | **14 (9%)** | **13 (8%)** | 12 (8%) |
| Severe | **0** | **1 (<1%)** | **0** | **2 (1%)** | **0** | 1 (1%) |
| Headache | **103 (45%)** | **92 (40%)** | **52 (39%)** | **64 (40%)** | **65 (42%)** | 63 (42%) |
| Severe | **4 (2%)** | **3 (1%)** | **2 (1%)** | **3 (2%)** | **5 (3%)** | 2 (1%) |
| Fatigue | **126 (56%)** | **121 (52%)** | **68 (51%)** | **76 (48%)** | **84 (55%)** | 68 (45%) |
| Severe | **7 (3%)** | **7 (3%)** | **3 (2%)** | **4 (3%)** | **4 (3%)** | 3 (2%) |
| Nausea | **42 (19%)** | **36 (16%)** | **17 (13%)** | **21 (13%)** | **28 (18%)** | 26 (17%) |
| Severe | **1 (<1%)** | **0** | **0** | **1 (1%)** | **0** | 0 |
| Loss of appetite | **41 (18%)** | **33 (14%)** | **22 (16%)** | **18 (11%)** | **28 (18%)** | 14 (9%) |
| Severe | **2 (1%)** | **3 (1%)** | **0** | **0** | **0** | 0 |
| Fever, ≥38°C | **5 (2%)** | **4 (2%)** | **1 (1%); N=133** | **3 (2%); N=158** | **5 (3%)** | 2 (1%) |
| M1 | N=214 | N=216 | N=128 | N=151 | **N=150** | **N=144** |
| Chills | 17 (8%) | 26 (12%) | 12 (9%) | 15 (10%) | **26 (17%)** | **32 (22%)** |
| Severe | 0 | 1 (<1%) | 0 | 0 | **1 (1%)** | **0** |
| Myalgia | 15 (7%) | 20 (9%) | 15 (12%) | 18 (12%) | **27 (18%)** | **37 (26%)** |
| Severe | 0 | 3 (1%) | 1 (1%) | 0 | **1 (1%)** | **1 (1%)** |
| Arthralgia | 7 (3%) | 11 (5%) | 9 (7%) | 7 (5%) | **10 (7%)** | **8 (6%)** |
| Severe | 0 | 0 | 1 (1%) | 1 (1%) | **0** | **0** |
| Headache | 67 (31%) | 47 (22%) | 36 (28%) | 41 (27%) | **70 (47%)** | **63 (44%)** |
| Severe | 1 (< 1%) | 2 (1%) | 2 (2%) | 2 (1%) | **3 (2%)** | **4 (3%)** |
| Fatigue | 61 (29%) | 64 (30%) | 40 (31%) | 49 (32%) | **64 (43%)** | **64 (44%)** |
| Severe | 0 | 3 (1%) | 2 (2%) | 0 | **4 (3%)** | **1 (1%)** |
| Nausea | 17 (8%) | 15 (7%) | 10 (8%) | 15 (10%) | **20 (13%)** | **26 (18%)** |
| Severe | 0 | 0 | 0 | 0 | **0** | **0** |
| Loss of appetite | 17 (8%) | 11 (5%) | 12 (9%) | 9 (6%) | **15 (10%)** | **19 (13%)** |
| Severe | 0 | 1 (<1%) | 0 | 0 | **0** | **2 (1%)** |
| Fever, ≥38°C | 2 (1%) | 6 (3%) | 2 (2%); N=127 | 3 (2%) | **4 (3%); N=149** | **9 (6%)** |
| M2 | **N=212** | **N=217** | N=125 | **N=150** | N=144 | N=138 |
| Chills | **33 (16%)** | **33 (15%)** | 7 (6%) | **22 (15%)** | 9 (6%) | 12 (9%) |
| Severe | **1 (< 1%)** | **1 (< 1%)** | 0 | **0** | 0 | 1 (1%) |
| Myalgia | **52 (25%)** | **40 (18%)** | 10 (8%) | **34 (23%)** | 13 (9%) | 13 (9%) |
| Severe | **2 (1%)** | **2 (1%)** | 0 | **2 (1%)** | 0 | 2 (1%) |
| Arthralgia | **16 (8%)** | **17 (8%)** | 3 (2%) | **11 (7%)** | 4 (3%) | 6 (4%) |
| Severe | **0** | **1 (< 1%)** | 0 | **0** | 0 | 0 |
| Headache | **90 (42%)** | **76 (35%)** | 28 (22%) | **53 (35%)** | 42 (29%) | 30 (22%) |
| Severe | **3 (1%)** | **4 (2%)** | 0 | **0** | 1 (1%) | 3 (2%) |
| Fatigue | **106 (50%)** | **96 (44%)** | 32 (26%) | **55 (37%)** | 44 (31%) | 32 (23%) |
| Severe | **4 (2%)** | **5 (2%)** | 0 | **1 (1%)** | 1 (1%) | 1 (1%) |
| Nausea | **35 (17%)** | **24 (11%)** | 8 (6%) | **21 (14%)** | 10 (7%) | 4 (3%) |
| Severe | **0** | **0** | 0 | **0** | 0 | 0 |
| Loss of appetite | **28 (13%)** | **28 (13%)** | 7 (6%) | **17 (11%)** | 7 (5%) | 11 (8%) |
| Severe | **1 (< 1%)** | **0** | 0 | **0** | 1 (1%) | 1 (1%) |
| Fever, ≥38°C | **6 (3%)** | **3 (1%)** | 0; N=124 | **4 (3%)** | 2 (1%); N=143 | 4 (3%) |
| M6 | N=206 | N=211 | **N=120** | **N=152** | N=139 | N=136 |
| Chills | 11 (5%) | 11 (5%) | **12 (10%)** | **31 (20%)** | 10 (7%) | 9 (7%) |
| Severe | 2 (1%) | 0 | **0** | **1 (1%)** | 0 | 0 |
| Myalgia | 17 (8%) | 20 (9%) | **26 (22%)** | **39 (26%)** | 11 (8%) | 13 (10%) |
| Severe | 1 (<1%) | 1 (<1%) | **2 (2%)** | **2 (1%)** | 1 (1%) | 0 |
| Arthralgia | 8 (4%) | 10 (5%) | **10 (8%)** | **18 (12%)** | 5 (4%) | 7 (5%) |
| Severe | 1 (<1%) | 0 | **0** | **2 (1%)** | 0 | 0 |
| Headache | 49 (24%) | 35 (17%) | **44 (37%)** | **52 (34%)** | 26 (19%) | 26 (19%) |
| Severe | 3 (1%) | 3 (1%) | **2 (2%)** | **4 (3%)** | 0 | 0 |
| Fatigue | 50 (24%) | 56 (27%) | **51 (43%)** | **73 (48%)** | 37 (27%) | 35 (26%) |
| Severe | 2 (1%) | 3 (1%) | **5 (4%)** | **3 (2%)** | 0 | 2 (1%) |
| Nausea | 21 (10%) | 13 (6%) | **11 (9%)** | **23 (15%)** | 9 (6%) | 14 (10%) |
| Severe | 1 (<1%) | 0 | **0** | **0** | 0 | 0 |
| Loss of appetite | 11 (5%) | 10 (5%) | **13 (11%)** | **23 (15%)** | 4 (3%) | 11 (8%) |
| Severe | 0 | 0 | **0** | **0** | 0 | 0 |
| Fever, ≥38°C | 0 | 2 (1%) | **3 (3%); N=119** | **7 (5%); N=151** | 0; N=138 | 0 |
| M12 | N=206 | N=206 | N=121 | N=147 | N=140 | **N=132** |
| Chills | 19 (9%) | 23 (11%) | 11 (9%) | 13 (9%) | 17 (12%) | **22 (17%)** |
| Severe | 0 | 0 | 1 (1%) | 0 | 0 | **0** |
| Myalgia | 16 (8%) | 26 (13%) | 12 (10%) | 19 (13%) | 19 (14%) | **21 (16%)** |
| Severe | 0 | 1 (<1%) | 0 | 1 (1%) | 0 | **1 (1%)** |
| Arthralgia | 8 (4%) | 9 (4%) | 6 (5%) | 11 (7%) | 8 (6%) | **11 (8%)** |
| Severe | 0 | 0 | 0 | 0 | 0 | **0** |
| Headache | 64 (31%) | 40 (19%) | 33 (27%) | 37 (25%) | 33 (24%) | **52 (39%)** |
| Severe | 1 (<1%) | 1 (<1%) | 1 (1%) | 2 (1%) | 2 (1%) | **2 (2%)** |
| Fatigue | 62 (30%) | 59 (29%) | 41 (34%) | 46 (31%) | 37 (26%) | **51 (39%)** |
| Severe | 1 (<1%) | 2 (1%) | 0 | 3 (2%) | 4 (3%) | **6 (5%)** |
| Nausea | 16 (8%) | 15 (7%) | 11 (9%) | 12 (8%) | 8 (6%) | **21 (16%)** |
| Severe | 0 | 0 | 0 | 0 | 0 | **0** |
| Loss of appetite | 18 (9%) | 14 (7%) | 9 (7%) | 7 (5%) | 9 (6%) | **22 (17%)** |
| Severe | 0 | 0 | 0 | 0 | 1 (1%) | **0** |
| Fever, ≥38°C | 5 (2%) | 1 (<1%) | 0; N=120 | 1 (1%) | 2 (1%); N=139 | **3 (2%)** |
| 4CMenB, 4-component meningococcal serogroup B vaccine; MenABCWY, pentavalent meningococcal serogroup A, B, C, W and Y vaccine; AE, adverse event; N, number of participants; M, month. Data in bold are associated with a meningococcal vaccination. | | | | | | |

# Table S4. Unsolicited, serious and medically-attended adverse events reported in the primary and extension studies

|  |  |  |  | Number (%) of participants with AEs | | | | | |
| --- | --- | --- | --- | --- | --- | --- | --- | --- | --- |
| Primary study |  |  |  | 4CMenB-0-2 | MenABCWY-0-2 | MenABCWY-0-6 | MenABCWY-0-2-6 | MenABCWY-0-1 | MenABCWY-0-11 |
|  |  |  | N | 221 | 228 | 129 | 157 | 151 | 147 |
|  |  |  | Unsolicited AEs | | | | | | |
|  |  |  | After any vaccination | | | | | | |
|  |  |  | Any | 146 (66) | 148 (65) | 76 (59) | 146 (66) | 93 (62) | 97 (66) |
|  |  |  | Related | 25 (11) | 31 (14) | 16 (12) | 27 (17) | 23 (15) | 18 (12) |
|  |  |  | Within 30 days post-MenABCWY vaccination | | | | | | |
|  |  |  | Any | 108 (49) | 109 (48) | 56 (43) | 94 (60) | 66 (44) | 58 (41)^a^ |
|  |  |  | Related | 18 (8) | 24 (11) | 13 (10) | 24 (15) | 18 (12) | 12 (8)^a^ |
|  |  |  | Serious AEs | | | | | | |
|  |  |  | Any | 9 (4) | 3 (1) | 7 (5) | 6 (4) | 8 (5) | 2 (1) |
|  |  |  | Related | 0 | 1 (<1) | 0 | 0 | 1 (1) | 0 |
|  |  |  | MAAEs | | | | | | |
|  |  |  | Any | 103 (47) | 97 (43) | 57 (44) | 79 (50) | 68 (45) | 67 (46) |
|  |  |  | Related | 5 (2) | 4 (2) | 2 (2) | 2 (1) | 4 (3) | 1 (1) |
|  |  |  | Deaths | 0 | 0 | 0 | 0 | 0 | 0 |
| Extension study |  |  |  | 4CMenB-0-2 | MenABCWY-0-2 | MenABCWY-0-6 | MenABCWY-0-2-6 | Naïve-4CMenB | Naïve-MenABCWY |
|  |  |  | N | 126 | 127 | 74 | 77 | 101 | 99 |
|  |  |  | Unsolicited AEs | | | | | | |
|  |  |  | Any | 37 (29) | 35 (28) | 15 (20) | 15 (19) | 45 (45) | 49 (49) |
|  |  |  | Related | 5 (4) | 7 (6) | 2 (3) | 5 (6) | 15 (15) | 16 (16) |
|  |  |  | SAEs | 2 (2) | 0 | 0 | 0 | 1 (1) | 0 |
|  |  |  | MAAEs | 20 (16) | 18 (14) | 8 (11) | 7 (9) | 21 (21) | 24 (24) |

AE, adverse event; 4CMenB, 4-component meningococcal serogroup B vaccine; MenABCWY, pentavalent meningococcal serogroup A, B, C, W and Y vaccine; N, number of participants; SAE, serious AE; MAAE, medically-attended AE.

^a^ N=142.

# Table S5. Solicited adverse events in the extension study

|  | Number (%) of participants | | | | | |
| --- | --- | --- | --- | --- | --- | --- |
|  | 4CMenB-0-2 | MenABCWY-0-2 | MenABCWY-0-6 | MenABCWY-0-2-6 | Naïve-4CMenB | Naïve-MenABCWY |
| **Local solicited AEs** | | | | | | |
| E-M0 | N=126 | N=127 | N=74 | N=77 | N=101 | N=99 |
| Erythema | 22 (17%) | 22 (17%) | 14 (19%) | 10 (13%) | 10 (10%) | 12 (12%) |
| Severe | 2 (2%) | 0 | 0 | 0 | 0 | 1 (1%) |
| Induration | 20 (16%) | 18 (14%) | 6 (8%) | 14 (18%) | 10 (10%) | 15 (15%) |
| Severe | 1 (1%) | 0 | 0 | 0 | 0 | 1 (1%) |
| Pain | 115 (91%) | 108 (85%) | 64 (86%) | 67 (87%) | 93 (92%) | 94 (95%) |
| Severe | 13 (10%) | 6 (5%) | 3 (4%) | 0 | 3 (3%) | 4 (4%) |
| E-M2 |  |  |  |  | N=101 | N=98 |
| Erythema |  |  |  |  | 14 (14%) | 16 (16%) |
| Severe |  |  |  |  | 1 (1%) | 2 (2%) |
| Induration |  |  |  |  | 8 (8%) | 14 (14%) |
| Severe |  |  |  |  | 1 (1%) | 3 (3%) |
| Pain |  |  |  |  | 84 (83%) | 88 (90%) |
| Severe |  |  |  |  | 4 (4%) | 3 (3%) |
| **General solicited AEs** | | | | | | |
| E-M0 | N=126 | N=127 | N=74 | N=77 | N=101 | N=99 |
| Chills | 27 (21%) | 23 (18%) | 12 (16%) | 8 (10%) | 18 (18%) | 22 (22%) |
| Severe | 1 (1%) | 1 (1%) | 0 | 0 | 1 (1%) | 1 (1%) |
| Myalgia | 22 (17%) | 26 (20%) | 14 (19%) | 13 (17%) | 11 (11%) | 14 (14%) |
| Severe | 2 (2%) | 1 (1%) | 1 (1%) | 0 | 0 | 2 (2%) |
| Arthralgia | 15 (12%) | 12 (9%) | 9 (12%) | 5 (6%) | 8 (8%) | 12 (12%) |
| Severe | 2 (2%) | 0 | 1 (1%) | 0 | 0 | 0 |
| Headache | 61 (48%) | 55 (43%) | 28 (38%) | 19 (25%) | 31 (31%) | 47 (47%) |
| Severe | 2 (2%) | 1 (1%) | 1 (1%) | 1 (1%) | 0 | 1 (1%) |
| Fatigue | 64 51%) | 58 (46%) | 31 (42%) | 33 (43%) | 45 (45%) | 48 (48%) |
| Severe | 3 (2%) | 2 (2%) | 3 (4%) | 0 | 1 (1%) | 4 (4%) |
| Nausea | 24 (19%) | 13 (10%) | 8 (11%) | 8 (10%) | 12 (12%) | 10 (10%) |
| Severe | 1 (1%) | 0 | 0 | 1 (1%) | 1 (1%) | 1 (1%) |
| Loss of appetite | 20 (16%) | 15 (12%) | 7 (9%) | 9 (12%) | 12 (12%) | 13 (13%) |
| Severe | 1 (1%) | 1 (1%) | 0 | 2 (3%) | 1 (1%) | 0 |
| Fever, ≥38°C | 6 (5%) | 8 (6%) | 1 (1%) | 3 (4%) | 4 (4%) | 1 (1%) |
| E-M2 |  |  |  |  | N=101 | N=98 |
| Chills |  |  |  |  | 19 (19%) | 9 (9%) |
| Severe |  |  |  |  | 1 (1%) | 1 (1%) |
| Myalgia |  |  |  |  | 15 (15%) | 16 (16%) |
| Severe |  |  |  |  | 1 (1%) | 0 |
| Arthralgia |  |  |  |  | 12 (12%) | 9 (9%) |
| Severe |  |  |  |  | 0 | 0 |
| Headache |  |  |  |  | 34 (34%) | 39 (40%) |
| Severe |  |  |  |  | 4 (4%) | 0 |
| Fatigue |  |  |  |  | 44 (44%) | 34 (35%) |
| Severe |  |  |  |  | 2 (2%) | 1 (1%) |
| Nausea |  |  |  |  | 11 (11%) | 13 (13%) |
| Severe |  |  |  |  | 2 (2%) | 0 |
| Loss of appetite |  |  |  |  | 9 (9%) | 15 (15%) |
| Severe |  |  |  |  | 1 (1%) | 0 |
| Fever, ≥38°C |  |  |  |  | 2 (2%) | 2 (2%) |

4CMenB, 4-component meningococcal serogroup B vaccine; MenABCWY, pentavalent meningococcal serogroup A, B, C, W and Y vaccine; AE, adverse event; N, number of participants; E-M, study month in the extension study.

# Figure S1. Percentage of participants with 4-fold increase in hSBA titers from pre-vaccination levels for fHbp, NadA, PorA and NHBA in the primary and extension studies (adapted full analysis set)


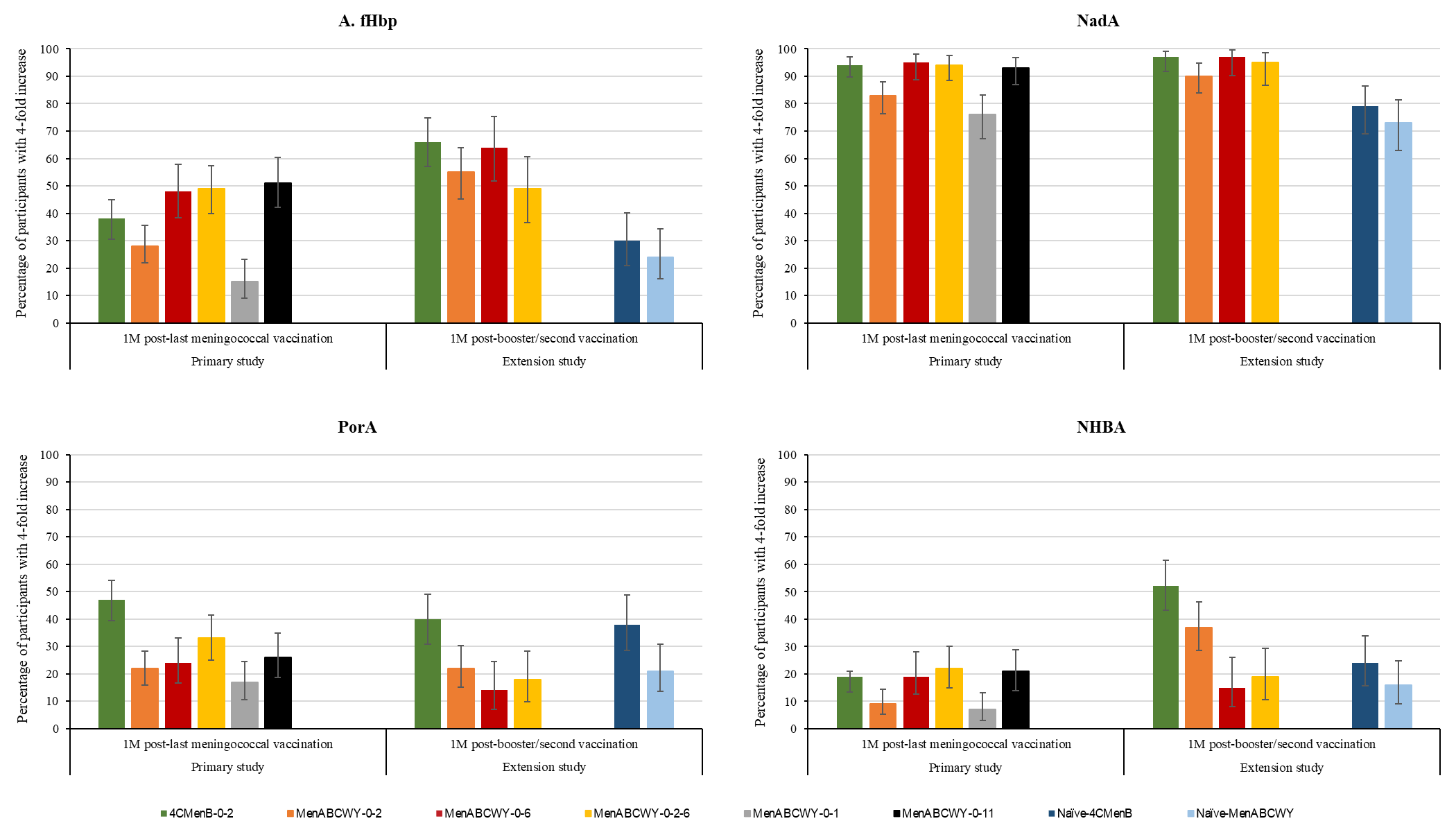


hSBA, serum bactericidal assay using human complement; fHbp, factor H binding protein; M, month; NadA*, Neisseria* adhesin; PorA, porin A; NHBA, Neisserial heparin binding antigen; 4CMenB, 4-component meningococcal serogroup B vaccine; MenABCWY, pentavalent meningococcal serogroup A, B, C, W and Y vaccine.

Error bars represent 95% confidence intervals.

A 4-fold titer rise was defined as a post-vaccination hSBA ≥4 LLQ for participants with pre-vaccination hSBA titers <LLOQ and) an increase of ≥4 times the pre-vaccination hSBA titer for participants with pre-vaccination hSBA titers ≥LLOQ (LLOQ, lower limit of quantitation [8.0 for fHbp, 8.6 for NadA, 8.2 for PorA and 8.9 for NHBA])

# Figure S2. Percentage of participants with 4-fold increase in hSBA titers from pre-vaccination levels for meningococcal serogroups A, C, W and Y in the primary and extension studies (adapted full analysis set)

**
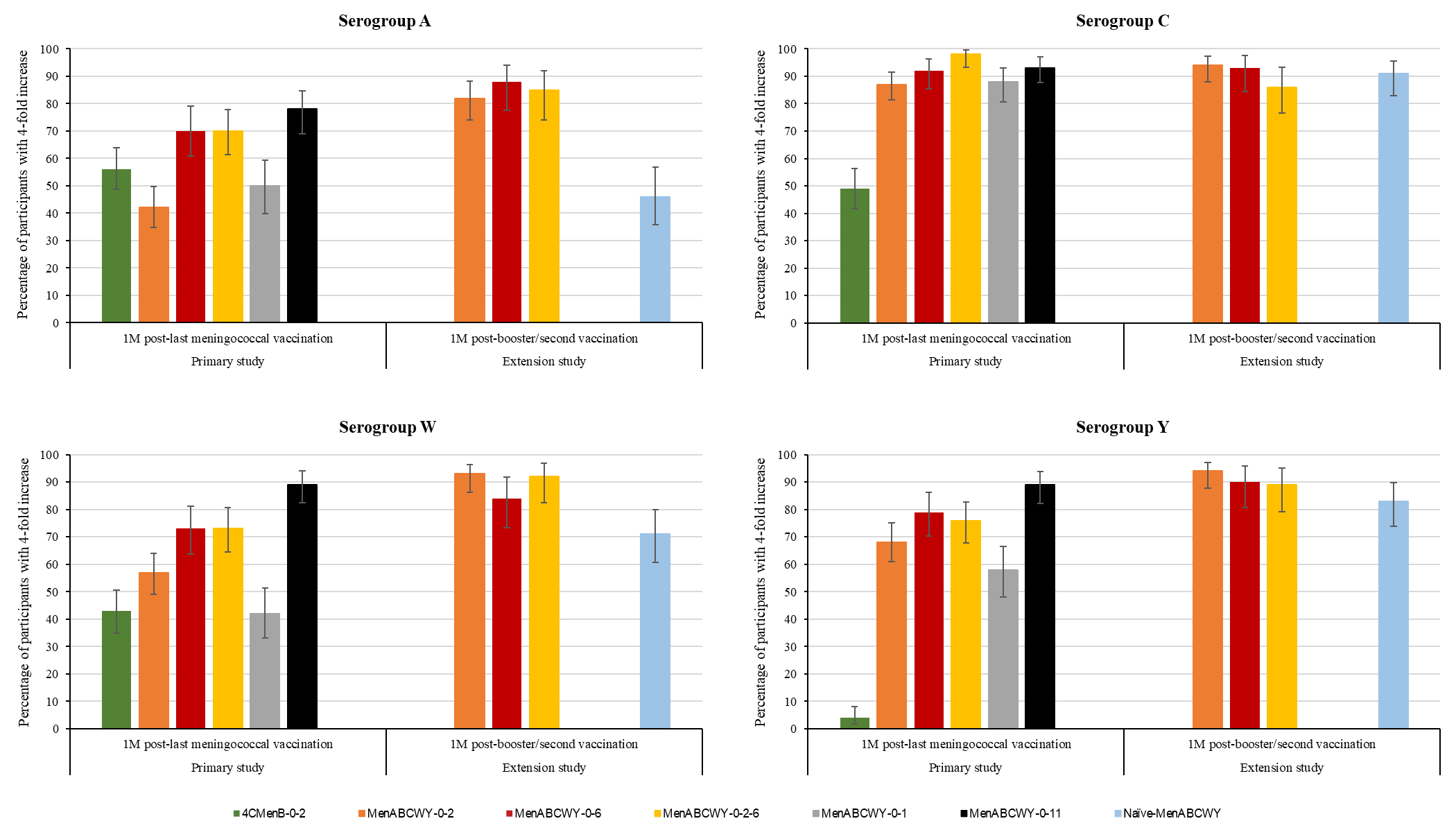
**

hSBA, serum bactericidal assay using human complement; 4CMenB, 4-component meningococcal serogroup B vaccine; MenABCWY, pentavalent meningococcal serogroup A, B, C, W and Y vaccine.

Error bars represent 95% confidence intervals.

A 4-fold titer rise was defined as a post-vaccination hSBA ≥4 LLQ for participants with pre-vaccination hSBA titers <LLOQ and an increase of ≥4 times the pre-vaccination hSBA titer for participants with pre-vaccination hSBA titers ≥LLOQ (LLOQ, lower limit of quantitation [22.7 for serogroup A, 5.2 for serogroup C, 39.8 for serogroup W and 14.7 for serogroup Y]).
